# ERP: An elastic resource provisioning approach for cloud applications

**DOI:** 10.1371/journal.pone.0216067

**Published:** 2019-04-26

**Authors:** Danqing Feng, Zhibo Wu, DeCheng Zuo, Zhan Zhang

**Affiliations:** 1 Computer science and Technology, Harbin Institute of Technology, Harbin, China; 2 Computer science and Technology, Air Force Communication NCO Academy, DaLian, China; Northeast Electric Power University, CHINA

## Abstract

Elasticity is the key technique to provisioning resources dynamically in order to flexibly meet the users’ demand. Namely, the elasticity is aimed at meeting the demand at any time. However, the aforementioned approaches usually provision virtual machines (VMs) in a coarse-grained manner just by the CPU utilization. Actually, two or more elements are needed for the performance metric, including the CPU and the memory. It is challenging to determine a suitable threshold to efficiently scale the resources up or down. In this paper we present an elastic scaling framework that is implemented by the cloud layer model. First we propose the elastic resource provisioning (ERP) approach on the performance threshold. The proposed threshold is based on the Grey relational analysis (GRA) policy, including the CPU and the memory. Secondly, according to the fixed threshold, we scale up the resources from different granularities, such as in the physical machine level (PM-level) or virtual machine level (VM-level). In contrast, we scale down the resources and shut down the spare machines. Finally, we evaluate the effectiveness of the proposed approach in real workloads. The extensive experiments show that the ERP algorithm performs the elastic strategy efficiently by reducing the overhead and response time.

## Introduction

Cloud computing is popular in industry due to its ability to deliver on-demand resources according to a pay-as-you-go model [1]. Usually, three basic service models are included in cloud computing: Infrastructure as a Service (IaaS) [2], Platform as a Service (PaaS) [3] and Software as a Service (SaaS) [4]. Namely, SaaS provides access to complete applications as a service. PaaS provides a platform for developing other applications on top of it, such as the Google App Engine (GAE) and Azure. IaaS provides an environment to deploy the managed virtual machines. Technically, when the users submit the requests, the providers would provide the resources depending on the users’ demand [5–6]. As a key technique in cloud computing, the elasticity [7] has the ability to acquire and release the resources according to the users’ demand.

Generally, the providers implement an automatic provisioning approach via the virtualization technique [8]. Virtualization makes it possible to rapidly scale the resources up or down. The aforementioned approaches [9] present a reactive method, which is triggered by a certain threshold, such as CPU utilization or memory utilization. Actually, two or more thresholds [10] should be used as a performance metric. In addition, it is important to provision the correct amount of the resources efficiently using a suitable threshold. In fact, the fluctuating workload would lead to an overprovisioning state or an underprovisioning state. To avoid these problems, researchers usually use a predictive technique, such as the proactive method. These feasible predictive approaches, such as machine learning [11–12], Moving Average [13], and Auto-Regression [14], would track the dynamic resource requirement and effectively minimize the energy consumption. This predictive policy would quantify the requirement in advance in order to flexibly scale the resources up or down. However, it is a challenging issue to improve the accuracy of the predictive technique. Additionally, an estimation error would lead to an overprovisioning or underprovisioning state. When in the sudden workload, this predictive method is especially inaccurate. Thus, combining this with an automatic method and a proactive method would be more agile for provisioning the resources. For example, the Elastic VM architecture [15] provisions the resources dynamically to reduce the SLA violation. However, the elasticity is necessary to meet the users’ demand from different perspectives. Some researchers would take the performance metrics into consideration, such as the SLA [16] and the profit of the providers [17]. However, more metrics are used in the elasticity to evaluate the performance [18]. For example, from the purpose of the providers, they might consider more related elements. That is, they would seek to minimize the renting cost, the energy consumption and the Service Level Agreement (SLA) violation. In summary, the elasticity would be implemented for one or two purposes, such as saving energy [19–20] or reducing the cost [21–22]. However, it is difficult to make an elasticity solution by considering multiple objectives. To solve the mentioned issues, we propose the ERP approach to provision the resources by the performance threshold, including the CPU and the memory. According to the threshold, we would flexibly scale the resources up or down by considering multiple perspectives. From the perspective of the provider, the goal is aimed at minimizing the amount of the resources to reduce the energy consumption. From the perspective of the users, the goal is aimed at rapidly scaling the resources up or down. In brief, the ERP approach is aimed at maximizing the utilization and minimizing the SLA violation. Then, the main contributions would be summarized in the following.

First, this approach solves the suitable threshold to determine the users’ demand. We present the performance threshold by using the GRA method, which considers such multiobjectives as the CPU utilization and memory utilization. Meanwhile, it is instructed on the cloud layer model using the MAPE loop. Usually the MAPE loop includes four phases, such as Monitoring (M), Analysis (A), Planning (P) and Execution (E).

Second, this approach solves the issue of scaling the resources flexibly. According to the proposed threshold, we could efficiently scale the resources up or down. That is, we propose a fine-grained algorithm, which means to scale up the resources from the PM-level or the VM-level in order to flexibly meet the users’ demand.

Third, this approach solves the issue of reducing the overheads. When it is overprovisioned, we would shut down the extra machines to reduce the energy consumption via a simple predictive technique, such as the weighted moving average (WMA).

The remainder of the paper is described as listed below. Section 2 analyses the related literature on the elastic techniques in cloud computing. Section 3 presents the ERP framework based on the layer model. Section 4 provides the performance threshold via the cloud layer model. Section 5 presents the effective ERP algorithm, which would scale the resources up or down from different granularities. Section 6 proves the results by comparing them with the aforementioned approaches. Finally, section 7 draws conclusions and describes future development.

## Related work

Usually the elastic solution is implemented by scaling the resources in or out. By analyzing some related works, we would divide the elastic resource provisioning approaches into two major aspects, including automatic scaling methods [23] and elastic mechanisms on the predictive technique [24].

### Automatic scaling methods

In the automatic policy, the resources would be provisioned and released automatically according to the demand. Generally, the action is triggered by the fixed thresholds, such as the utilization. The common techniques are provided by Amazon and Scalr. However, they provision the resources only based on the utilization, when in fact more elements have taken effect. Additionally, its advantage is a kind of coarse-grained provisioning strategy to scale the virtual machines. When considering the fine-grained provisioning strategy, some researchers focus on the reactive methods by resizing the resources dynamically and minimizing the response time and executing cost in cloud computing. However, they focus more on the fine-grained scaling strategy, and less on multiple perspectives. Kingfisher [25] proposed an elastic mechanism to reduce the transition of time and cost. This approach exploits the available resources on the virtual machines to scale in or out, and uses an integer linear program formulation to optimize the cost. Leitner et al. [26] proposed the SLA-aware scheduling algorithm, which would reduce the request execution time. It presents a cost-efficient method to scale up from the perspective of the providers. In contrast, our approach considers more factors to formulate the threshold by the cloud layer model, such as CPU utilization, memory utilization, etc. Additionally, we aim to scale the resources by minimizing the renting cost and response time. This would shut down the spare machines from the perspective of saving the consumption. By analyzing the mentioned works, we determined that most recent elastic strategies focus on the horizontal elasticity. Therefore, it is important to scale the resources from different granularities, including horizontal elasticity [27] and vertical elasticity [28]. By considering the fine-grained elasticity, we present the ERP algorithm to scale up the resources in the PM-level or VM-level by the performance threshold. Moreover, when it is in overprovisioning, it would scale down the resources in the VM-level.

### Elastic mechanisms based on the prediction

In fact, elasticity is essential to meet a fluctuating workload, and it is necessary to determine the suitable amount of the resources in order to scale the resources. Actually, the proactive approaches are used to determine the next demand, such as the Autoregressive moving average model (ARMA) [29] and Holt winter [30]. These predictive techniques have the advantage of giving an accurate prediction value in the stable workload. However, these predictive techniques focus more on the accuracy, but ignore the complexity. Moreover, when a sudden workload appears it might be in estimation error. To reduce the complexity of the prediction algorithm, some techniques are used to determine the repetitive patterns and predict the next values. PRESS [31] is a predictive elasticity system that analyzes and extracts the workload patterns and provisions the resources automatically. The advantage of this policy is that improves the prediction accuracy, and it reduces the resource waste efficiently. However, it only makes emphasis on the overhead. CloudScale [32] is a system that automates the fine-grained resources in cloud computing infrastructures, determining the adaptive resources by the prediction. In addition, it integrates the dynamic CPU voltage scaling to saving the consumption by migration. This technique puts more emphasis on the proactive method based on the prediction, which would minimize the energy consumption and avoid the Service Level Object (SLO) violation. In fact, more elements should be taken into consideration. Hence, in our approach, we consider more elements, such as reducing the renting cost, energy consumption and SLA violation. Additionally, we increase or decrease the resources automatically from different granularities to meet the demand, including fine-grained scaling and coarse-grained scaling. Namely, when it is underprovisioning, our approach scales up the resources from different granularities by the performance threshold, such as in the PM-level or VM-level. In contrast, we scale down the VMs by the WMA predictive technique efficiently.

## Proposed approach

In this section, we present our proposed approach for the detailed description. Our approach is designed on the cloud layer model. That is, this policy is implemented to determine the performance threshold to flexibly scale the resources up or down. Additionally, the formulation of the performance threshold is presented in detail in the next section. Then the ERP framework is explained in detail in the following.

### Cloud layer model

**Fig 1:**
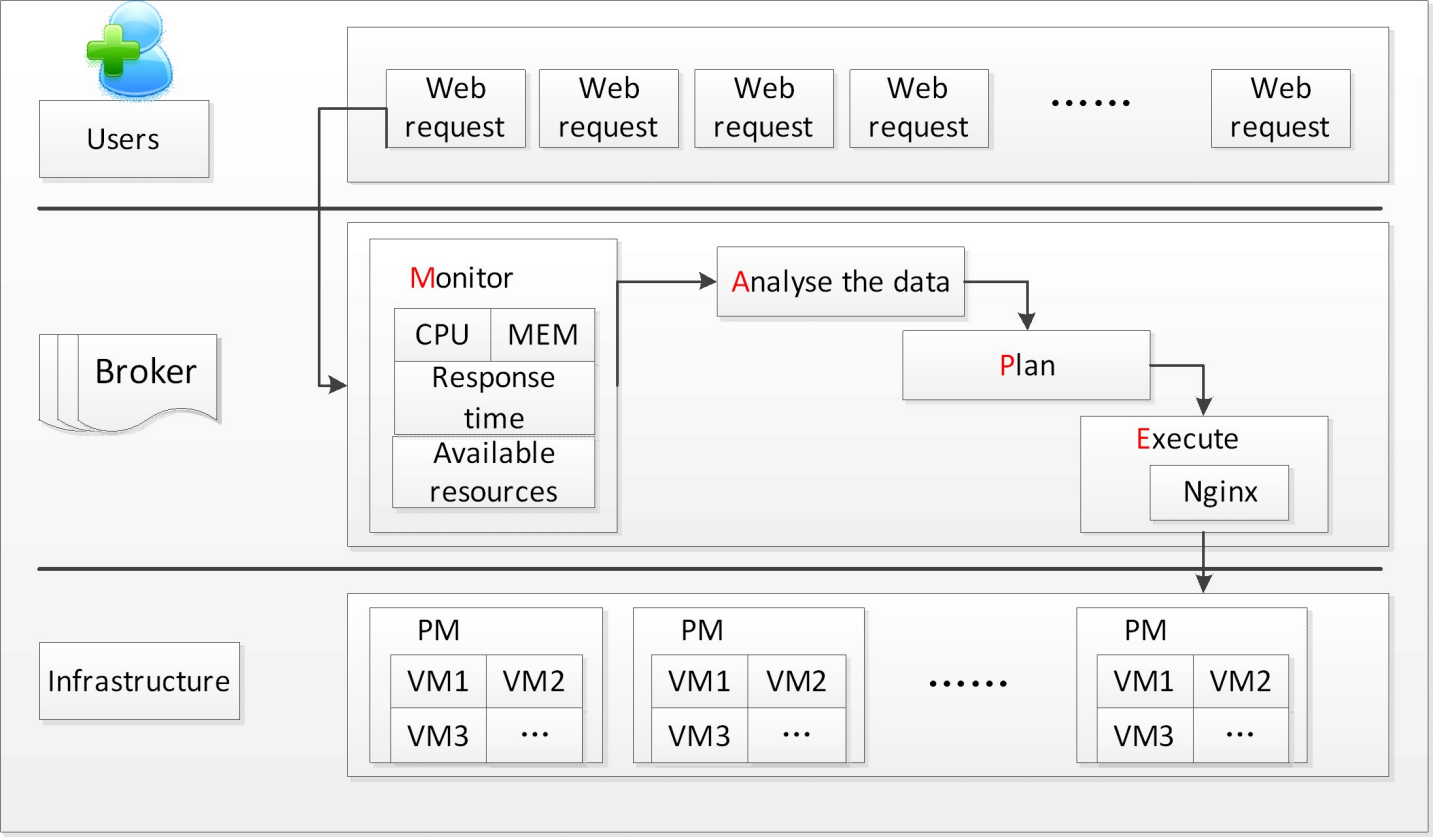
The process of resource allocation.

In this section, our approach describes a cloud layer model to scale the resources rapidly. The cloud layer model focuses more on the quantitative analysis, whereas the Delphi method [33] depends more on the subjective assessment. The ERP approach is implemented on the cloud layer model. The layer model is composed of three parts: SaaS, PaaS and IaaS. The SaaS determines a series of requests offered by the users. In the PaaS the broker is responsible for provisioning the infrastructure resources according to the users’ demandwhich is presented by the MAPE loop. In IaaS, the datacenter is composed of some PMs and VMs. The provider would provision the resources according to the requests. As depicted in Fig 1, the key components of the MAPE are described in detail as follows.

#### Monitor (M)

The monitoring component collects some metrics, such as the CPU utilization, memory utilization and some available resources. It monitors the information every five seconds. The key information is collected, aggregated and calculated by the performance model, which is described in detail in the next section.

#### Analyze (A)

The analyzing phase is responsible for analyzing the collected information. The obtained data is aggregated and calculated by the performance model, and we achieve the performance value to decide whether the scaling action is triggered. Moreover, we use the WMA predictive technique to determine the correct number of the servers and shut down the spare machines.

#### Plan (P)

This component is the core of the cloud layer model. According to the users’ demand, it implements the scaling strategy by minimizing the renting cost and reducing the energy consumption. Additionally, it would increase or decrease the resources by the performance threshold.

#### Execute (E)

In the executing phase, the Nginx load balancing server balances the web requests by provisioning the servers in the infrastructure. Since the VMs are hosted in the PMs, the provider would provision the resources according to the demand by using the proposed plan.

### Proposed framework

**Fig 2:**
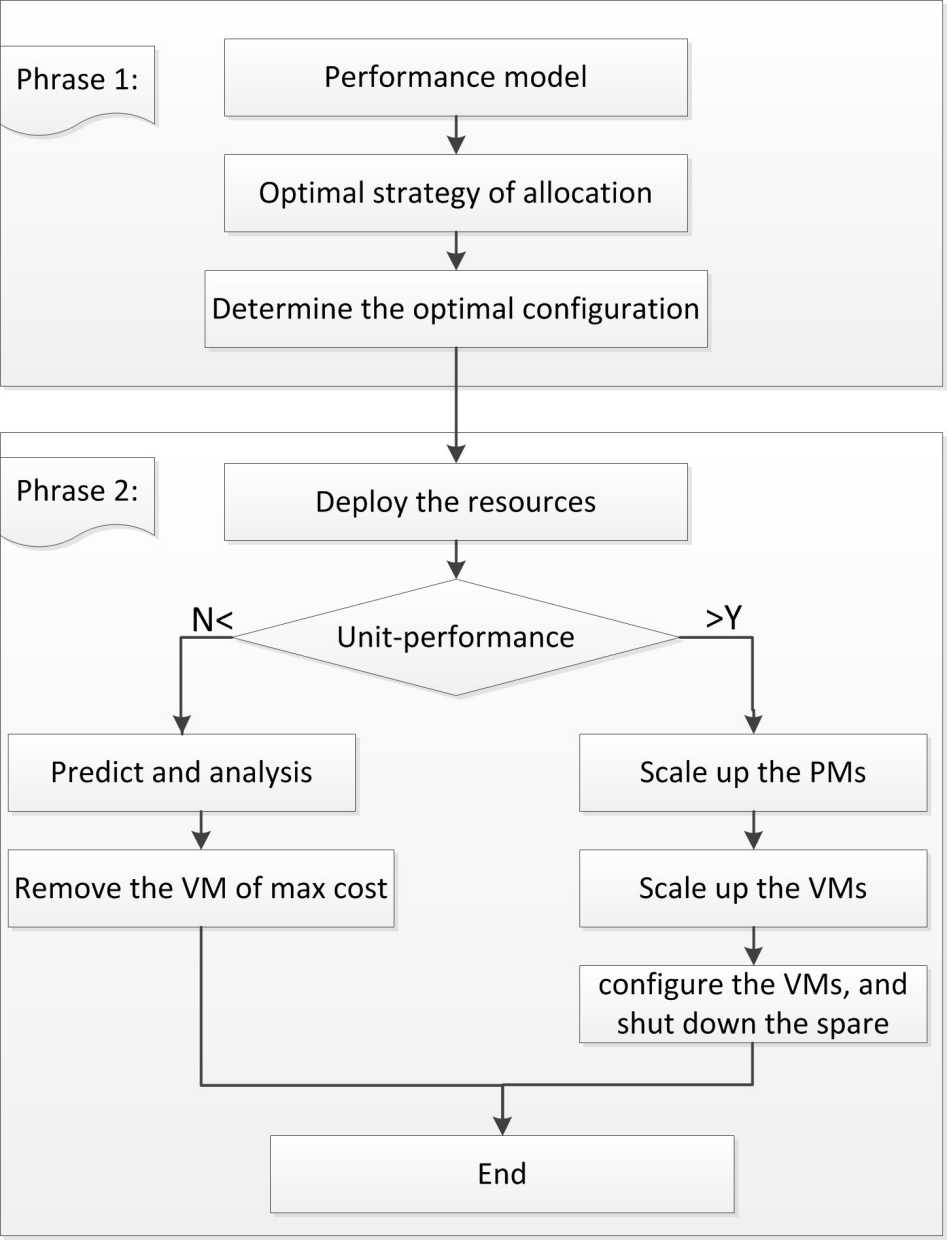
A framework of the entire system architecture.

In our approach we propose a novel framework to flexibly increase or decrease the resources aiming at minimizing the renting cost, energy consumption and response time, as illustrated in Fig 2. The ERP algorithm is mainly composed of two phases. In the first phase, the performance model constructs a baseline threshold, which is aggregated and calculated by the gathered data. From this the resources would be rapidly scaled up or down. In the second phase, the ERP algorithm is used to scale the resources by the performance threshold for the purpose of minimizing the renting cost and saving power consumption.

Then, we explain these two phases in detail. In the first step, the monitoring component monitors the CPU utilization, memory utilization, CPU clock speed and some available resources. We aggregate the gathered data to make a performance evaluation by the proposed cloud layer model. In the second step, we make a further description on the ERP approach. In the analyzing component, we scale the resources by the performance threshold. Actually, the planning phase may lead into two states, including an underprovisioning state or overprovisioning state. When it is in an underprovisioning state, we execute the action on increasing the resources at the PM-level. If it continues, we go on scaling up the resources at the VM-level. The PM-level scaling depends on the available resources in the same host. The VM-level scaling is based on the VMs hosted on the PMs. Additionally, the VM could come from the same PM or another PM. Otherwise, when it is in overprovisioning we scale down the resources by the prediction. Then the extra spared machines would be shut down by saving the energy consumption. Moreover, our approach implements the elastic scaling from different granularities with the consideration of minimizing the cost and the SLA violation.

## Performance threshold

In this section, we present a performance threshold on multiple elements. From this we would rapidly scale the resources up or down in cloud computing.

### TOPSIS and GRA policy

This policy presents a multicriteria threshold that takes five related criterion into account, as shown in Table 1. The criteria on the TOPSIS and GRA policy would include the cost type and benefit type. After the matrix is normalized, the TOPSIS method evaluates them by the positive ideal solution and negative ideal solution. Then, the GRA method makes the decision from less information and explores the system behavior by analyzing the related degree.

**Table 1:** The parameters of the ERP approach.

Parameter	Description	Type
CPU Cycle	The CPU cycle refers to the CPU Clock Speed.	Cost
RAM Capability	RAM Capability is the rest memory capability.	Cost
CPU%	The utilization of CPU is the CPU usage of the PM.	Benefit
RAM%	The utilization of RAM is the RAM usage of the PM.	Benefit
Availability	Supposing that the resource availability is higher than 90%.	Cost

Usually the information on the PMs is gathered every 5 seconds to form the decision matrix, as shown in Eq 1. The gathered data is described as depicted in Table 1. Then we construct and implement the performance threshold in detail as follows.

$$R=[\begin{array}{ccccc} r_{cycle}{}_{{}_{1}} & r_{restMem_{1}} & r_{cpu{\%}_{1}} & r_{mem{\%}_{1}} & r_{avail{\%}_{1}}\\ r_{cycle}{}_{{}_{2}} & r_{restMem_{2}} & r_{cpu{\%}_{2}} & r_{mem{\%}_{2}} & r_{avail{\%}_{2}}\\ r_{cycle}{}_{{}_{3}} & r_{restMem_{3}} & r_{cpu{\%}_{3}} & r_{mem{\%}_{3}} & r_{avail{\%}_{3}} \end{array}]$$ (1)

#### Normalization of the decision matrix

In the first step we normalize the decision matrix. Namely, the decision matrix is normalized by achieving the average value of every column as listed in Eq 2.

$$R=[\begin{array}{ccccc} \frac{r_{{{}_{cycle}}_{{}_{1}}}}{SUM_{{}_{cycle}}} & \frac{r_{restMem_{1}}}{SUM_{restMem}} & \frac{r_{cpu{\%}_{1}}}{SUM_{{}_{cpu\%}}} & \frac{r_{mem{\%}_{1}}}{SUM_{mem\%}} & \frac{r_{avail{\%}_{1}}}{SUM_{avail\%}}\\ \frac{r_{cycle2}}{SUM_{cycle}} & \frac{r_{restMem_{2}}}{SUM_{restMem}} & \frac{r_{cpu{\%}_{2}}}{SUM_{{}_{cpu\%}}} & \frac{r_{mem{\%}_{2}}}{SUM_{mem\%}} & \frac{r_{avail{\%}_{1}2}}{SUM_{avail\%}}\\ \frac{r_{{}_{cycle}3}}{SUM_{{}_{cycle}}} & \frac{r_{restMem_{2}}}{SUM_{restMem}} & \frac{r_{cou\%3}}{SUM_{cpu\%}} & \frac{r_{mem\%3}}{SUM_{mem\%}} & \frac{r_{avail\%3}}{SUM_{avail\%}} \end{array}]$$ (2)

#### Improved TOPSIS

This is the abbreviation of the Technique for Order Preference by Similarity to Ideal Solution (TOPSIS). The traditional TOPSIS method depends more on subjective weights, while the improved TOPSIS solutions depend more on key factors. In the second step, the ideal solution would be determined by Eq 3 and Eq 4. That is, for the cost type the ideal solutions are the smaller ones, and the negative solutions are the larger ones. It is the opposite situation for the benefit type. Then, we achieve the positive ideal solution and the negative ideal solution, respectively.

$$P_{j}^{+}=\left\{\left(\max\left({\tilde{r}}_{ij}\right)|i\in I\right),\left(\min\left({\tilde{r}}_{ij}\right)|i\in J\right)\right\}$$ (3)

$$P_{j}^{-}=\left\{\left(\min\left({\tilde{r}}_{ij}\right)|i\in I\right),\left(\max\left({\tilde{r}}_{ij}\right)|i\in J\right)\right\}$$ (4)

#### Grey relational analysis

Grey theory is an effective way to solve multiobjective decision problems in the engineering areas [34–35]. In the following step, we determine the difference between the comparative series *r*_*jk*_ and the standard series $P_{k}^{+}$ or $P_{k}^{-}$. Additionally, the distinguish coefficient *ρ* is usually 0.5, and is generally between [0, 1]. Then, the Grey relational coefficients *ς*^+^and *ς*^−^ are constructed by Eq 5 and Eq 6, respectively.

$$\varsigma^{+}(k)=\frac{\underset{j}{\min}\;\underset{k}{\min}|r_{jk}-P_{k}^{+}|+\rho \underset{j}{\max}\;\underset{k}{\max}|r_{jk}-P_{k}^{+}|}{|r_{jk}-P_{k}^{+}|+\rho \underset{j}{\max}\;\underset{k}{\max}|r_{jk}-P_{k}^{+}|}$$ (5)

$$\varsigma^{-}(k)=\frac{\underset{j}{\min}\;\underset{k}{\min}|r_{jk}-P_{k}^{-}|+\rho \underset{j}{\max}\;\underset{k}{\max}|r_{jk}-P_{k}^{-}|}{|r_{jk}-P_{k}^{-}|+\rho \underset{j}{\max}\;\underset{k}{\max}|r_{jk}-P_{k}^{-}|}$$ (6)

Actually, the weight coefficients are determined by the analytic hierarchy process (AHP) method [36]. Then we determine the degree of relation *r* on the weight coefficients *ω* by multiplying them by Grey relational coefficient *ς*(*k*). Additionally, the degree of relations *r*^+^ and *r*^−^ are formulated by Eqs 7 and 8, respectively.

$$r^{+}=\sum_{k=1}^{m}\omega_{k}\varsigma_{}^{+}(k)$$ (7)

$$r^{-}=\sum_{k=1}^{m}\omega_{k}\varsigma_{}^{-}(k)$$ (8)

Then we formulate the relative closeness coefficient *u*^+^ to the ideal solution by Eq 9, which is implemented on the ideal relational coefficient *r*^+^ divided by the sum of the positive relational coefficient *r*^+^ and negative relational coefficient *r*^−^.

$$u^{+}=\frac{r^{+}}{r^{+}+r^{-}}$$ (9)

### Performance model

The performance threshold is constructed by the entropy method [37], which is an effective method to calculate the deviation degree. The smaller the entropy value is, the better the performance is. Similarly, the larger entropy value is, the worse the performance is. Therefore, we determine the performance threshold by the entropy method, which is listed as in Eq 10.

$$\Delta P=-\log_{2}(\frac{P_{2}}{P_{1}})=-\log_{2}(\frac{u_{2}{}^{+}}{u_{\max}}/\frac{u_{1}{}^{+}}{u_{\max}})=-\log_{2}(\frac{u_{2}^{+}}{u_{1}{}^{+}})$$ (10)

Where Δ*P* is the performance threshold by the entropy method, *P*_1_ is the probability before the demand varies, and *P*_2_ is the probability after the demand varies. Additionally, the probability is constructed on the ideal relational coefficient *u*^+^ divided by the max relational coefficient *u*_max_.

In the scheduling, a current performance value below 0.1 denotes a better performance environment. When it is above 3, it denotes a poor performance environment [38–39]. In fact, a normal value is between 0.1 and 3, which is described in Table 2. In our experiments, when the performance value is lower than 0.1, we would scale down the servers. Then, we set 0.1 as the lower threshold *P*_*d*_. When the value is greater than 0.2, we would scale up the servers for the purpose of reducing the response time by reserving slightly more resources. Then, we set 0.2 as the upper threshold *P*_*u*_.

**Table 2:** The description of the service-level.Scope.

	Service Level	Rule and Action
<0.1	Better-Level	Scaling down the servers
[0.1,0.9]	Normal-Level	In the normal statement
[0.9,3]	Worse-Level	In the worse statement
>3	Worst-Level	In the paralysis statement

## The ERP algorithm

In this section we describe the ERP algorithm to scale the resources from different granularities according to the users’ demand.

### ERP algorithm

To provision the resources flexibly, we first discuss some related definitions on the elasticity, such as the resilience and scalability. Next, we define them and clarify the difference between them. **Scalability** means to the ability of the system to deal with an increasing amount of the servers in a capable manner. However, it focuses more on the increasing ability, and less on the response time. **Resilience** means to provision the resources rapidly in a flexible way. Elastic scheduling refers to two core conditions, including the time and speed [40]. In this paper, we define an elastic scheme *S*, which is represented as *S* = (*clock*,*U*_*cpu*%_,*U*_*mem*%_,*P*_*u*_,*P*_*d*_), where *clock* is the CPU cycle, *U*_*cpu*%_ and *U*_*mem*%_ are the CPU utilization and the memory utilization, respectively, which are gathered by monitoring the system, and *P*_*u*_ and *P*_*d*_ are the upper and lower thresholds, respectively. In brief, the main algorithm (refer to Algorithm 1) provisions the resources rapidly via the MAPE loop. In the monitoring and analyzing components, some key elements are collected to determine the performance threshold. Then in the planning and executing components, the elastic scheme would scale the resources by the performance threshold. To make the ERP algorithm understood for the further step, Table 3 lists the main parameters of the ERP algorithm as below.

**Table 3:** The summary of the notations in the ERP algorithm.

Symbol	Description	Relation
PM	Physical machine	Related resources
VM	Virtual machine	Related resource
P	The current performance value	Calculated by the monitoring system
*P*_*u*_	The upper performance threshold	Determined by the experiments
*P*_*d*_	The lower performance threshold	Determined by the experiments
*u*_*cpu*%_	The CPU utilization of the VM	Monitoring system
*u*_*mem*%_	The memory utilization of the VM	Monitoring system
*c*(*p*_*i*_)	The renting cost of the PM	Cloud providers
*c*(*v*)	The renting cost of the VM	Cloud providers

Next, the ERP algorithm is described in detail. It implements an elastic resource provisioning approach in the datacenter. This algorithm takes the performance threshold as the baseline to scale the resources up or down. At first, the monitoring component would collect and gather the information as listed in Table 1 (lines 1–2) every few minutes. In fact, the ERP algorithm would increase or decrease the resources to meet the users’ demand. When the performance value *P* is larger than the upper threshold *P*_*u*_, the algorithm would be triggered to scale up the servers (SUS) (lines 4–5).In contrast, once the current performance value is below the threshold *P*_*d*_, the scaling down the servers (SDS) algorithm is triggered (lines 6–7).

**Algorithm 1**. **ERP (Elastic resource provisioning)**

1: Initialization: Server, P

2: while (the allocation is deploying)

3: monitor the performance value P

4: if P > *P*_*u*_

5:     Scaling up the servers (SUS)

6: else if P < *P*_*d*_

      Scaling down the servers (SDS)

7: End

The proposed ERP algorithm has included two aspects. First, the scaling up the servers (SUS) algorithm proposes a scaling method that is based on different granularities. That is, we scale up the VMs in the same available PMs or from some different PMs. Second, the scaling down the servers (SDS) algorithm presents the approach to shut down the extra machines.

### The SUS algorithm

The SUS algorithm is intended to scale up the resources in a flexible way, including from the PM-level or VM-level. The SUS algorithm is described by Algorithm 2. The monitoring component collects some metrics related to the resources (lines 1–4). If the performance evaluation reaches the upper threshold *P*_*u*_, it scales up more available resources on the PM (lines 5–7). When the updated performance value continues past on the upper threshold *P*_*u*_, we would provision slightly more resources (lines 8–10). Additionally, the VMs might come from different PMs.

**Algorithm 2**: **SUS (Scaling up the servers)**

1: Begin

2: Initialization: Server, P

3: while (the allocation is deploying)

4: monitor the performance value P

5: if P > *P*_*u*_

6:     Scaling up the PMs

7:     update the performance value P

8: while (P > *P*_*u*_)

9:     Scaling up the VMs

10:     update the performance value P

11: End

### The PLI algorithm

The purpose of the PM-Level increasing (PLI) algorithm is to increase the VMs on the available PMs (refer to Algorithm 3). Then we explore the PLI algorithm in detail. The monitoring component aggregates the information and calculates the performance value (lines 1–3). Once the triggered action appears we scale up the residual resources on the available PMs. Then we would choose the PMs aimed at minimizing the renting cost (lines 4–7). Additionally, the cost function is described by Eqs 11 and 12. Finally, it updates the performance value (line 8).

**Algorithm 3**: **PLI (PM-level increasing)**

1: Begin

2: Initialization: Server, P

3: Calculating the performance value

4: while (P > *P*_*u*_)

5:     if PM is available

6:     select the min cost PM to increase

7:     update the performance value

8: End

In this phase, Eq 11 is aimed at minimizing the renting cost, where *u*_*cpu*%_ presents the CPU utilization of the VM. The binary variable *v*_*j*_ indicates whether or not the VM is selected, and the binary variable *p*_*i*_ indicates whether or not the PM is selected. The parameter *m* is responsible for the amount of VMs hosted on the current host, and *c*(*p*_*i*_) is the expending cost of the current host.

$$Minimize\;p_{i}•\sum_{j=1}^{m}v_{j}•(1-u_{cpu\%})•c(p_{i})/\sum_{j=1}^{m}v_{j}$$ (11)

$$\begin{array}{l} subject\;to:\\ \sum_{j=1}^{m}v_{j}^{}\leq m,v_{j}^{}\in \{0,1\},P_{i}^{}\in \{0,1\} \end{array}$$ (12)

### The VLI algorithm

In this section, we propose the VM-level increasing (VLI) algorithm (refer to Algorithm 4) to continue increasing the resources to meet the fluctuating demand. It consists of three parts: monitoring the component, increasing the resources and updating the state. Then we describe the algorithm 4 in detail. First, the monitoring component gathers the information to calculate the performance value (lines 1–3). Second, we choose the suitable VMs to increase (lines 4–5), which would determine minimizing the expending cost by Eq 13. That is, we calculate the remaining utilization by the CPU and the memory. We implement the cost in Eq 13 by multiplying the remaining utilization by the single VM renting cost. The purpose of the function is to achieve the VM with a minimum cost, where the binary variable *v*_*j*_ indicates whether or not the VM is selected in Eq 14. Then we make a global search to find a suitable VM to increase. Finally, we update the state and calculate the performance value (lines 6–7).

**Algorithm 4**: **VLI (VM-level increasing)**

1: Begin

2: Initialization: Server, P

3: Calculating the performance value

4: while (P > *P*_*u*_)

5:     select the min cost VM to increase

6:     update the performance value

7: End

$$C_{vm}=\arg\min(v_{j}(1-u_{cpu\%})•(1-u_{mem\%})•c_{}(v))$$ (13)

$$j\in \{1,2,\ldots ,m\},v_{j}\in \{0,1\}$$ (14)

### The SDS algorithm

The aforementioned algorithms (refer to Algorithms 2–4) implement increasing the resources from a different granularity according to the users’ demand. In this section, the Scaling-down servers (SDS) algorithm is described for the detailed steps. In the first step we monitor the component and gather some information to achieve the performance threshold (lines 1–3). Once the SDS algorithm is triggered we would scale down the resources. Then we select the extra machines to shut down for the purpose of minimizing the cost (lines 4–5). Hence, we shut down the machines that occupy the maximum expending cost. Eq 15 is as listed below. Finally, we update the state and determine the current performance threshold (lines 6–7).

$$C_{{}_{vm}}^{'}=\arg\max(v_{j}(1-u_{cpu\%})•(1-u_{mem\%})•c(v))$$ (15)

**Algorithm 5**: **SDS (Scaling-down servers)**

1. Begin

2. Initialization: Server, P

3. Calculating the performance value

4. while (P < *P*_*d*_)

5.     select the max cost VM to decrease

6.     update the performance value

7. End

## Experiments

In this section, we implement the elastic resource allocation strategy based on the performance criterion. Meanwhile, the proposed approach proves that it is appropriate for meeting the demand in different kinds of workloads. In addition, this approach considers both reducing the renting cost and improving the utilization.

### Environment setup

We use the CloudStack platform and simulated real-world workloads to evaluate the ERP approach. We deploy a cluster composed of ten PMs. One installs the CloudStack platform. The other nine PMs use Xenserver as the management nodes (2.20GHz Intel(R) Xeon(R) 8 CPU, 8 G memory, running CenOs 6.9). We create 27 VMs (1 VCPU, 1 G memory, running CenOs 6.9) in the cluster. Then, the database is run off MySQL. When the workload is fluctuating, the Nginx has the function off balancing the servers. All the configuration information is listed in Table 4.

**Table 4:** Experiment environment.

Software	The configure
CloudStack	CloudStack 4.5
CentOS	CentOS 6.9
JDK	jdk1.8
MySQL	mysql 5.1
Nginx	nginx/1.8.0

**Fig 3:**
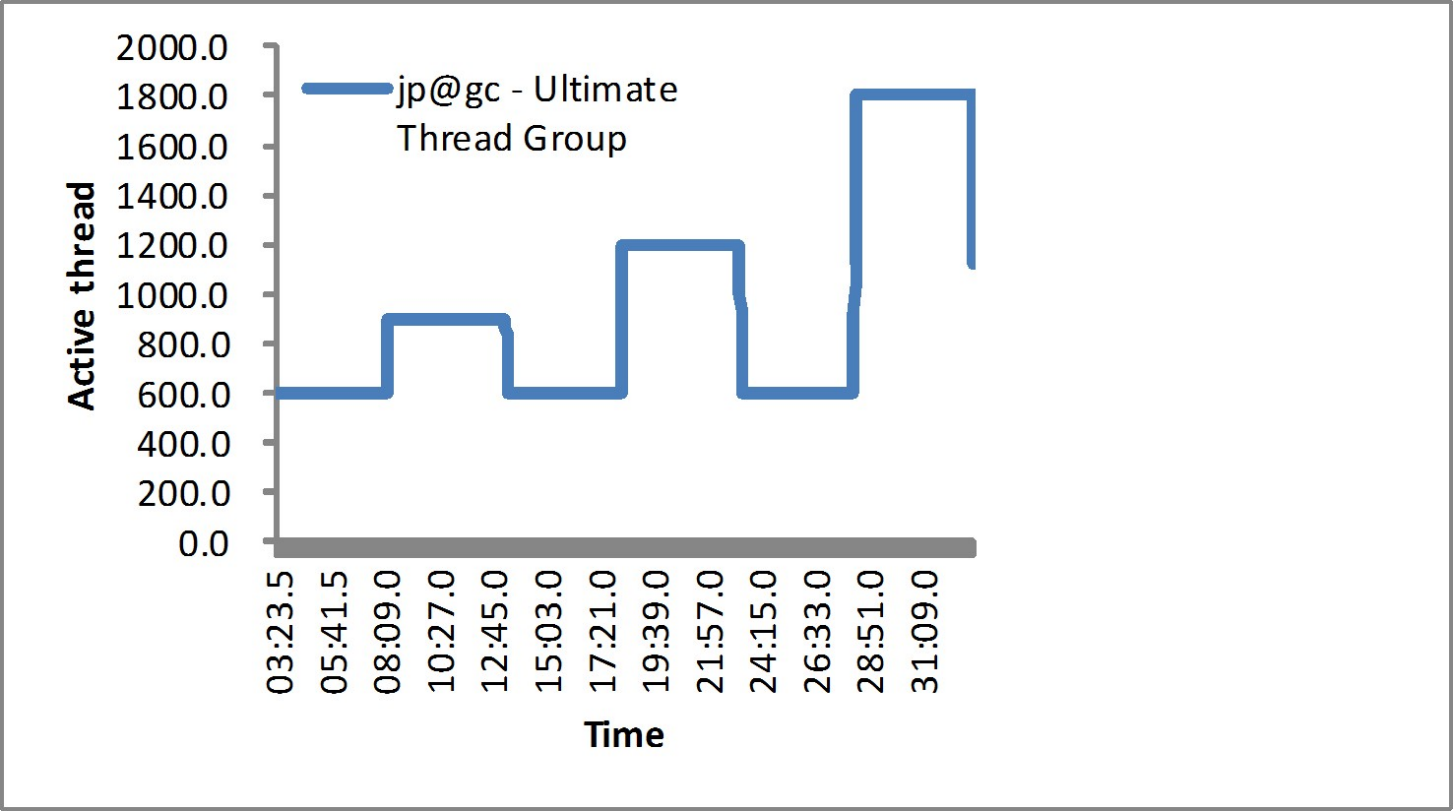
Active threads in the first experiment.

**Fig 4:**
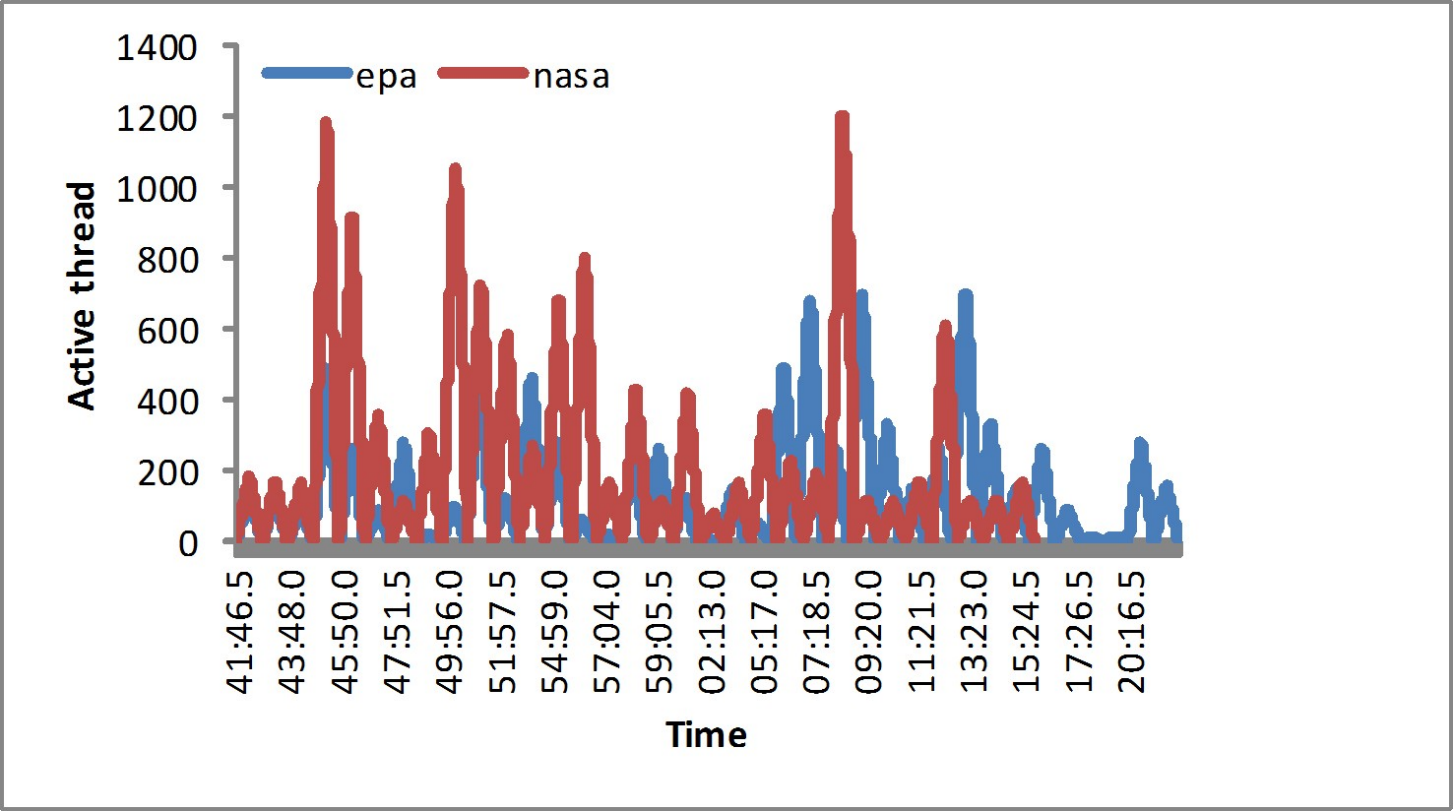
Real EPA and NASA tests in the second experiment.

To evaluate the proposed approach, we design two kinds of workloads: synthetic workloads and real-world workloads. We use the Jmeter to generate the requests based on the TPC benchmark. First, the synthetic workload would vary from the users’ demand. The fluctuating process of the workload is described as below. The load generator would implement 600, 900, 600, 1200, 600, and 1800 users, which is shown in Fig 3, which lasts for over 30 minutes. Second, the simulated real-world workload is extracted from the EPA and NASA traces [41]. The two kinds of real-world workload traces are generated as shown in Fig 4. Additionally, the monitoring service is implemented by the Jmeter plugins, such as monitoring the response time, CPU utilization or memory utilization. The experiment would last for over 40 minutes.

### Evaluation metric

In the experiments, we consider some performance indicators as the metrics, such as the renting cost, energy consumption, resource utilization and SLA violation.

#### The cost

This metric might be measured by the reserved and on-demand VMs. For example, the basic unit of the CPU is set at 1 GB in Aliyu. It is charged 0.059 ¥/hour in the reserved plan and 0.28 ¥/hour for the on-demand plan. The renting cost is defined in Eq 16, where *C*^*r*^ and *C*^*o*^ are responsible for the renting cost in the reserved or on-demand plan, respectively. Then in the scheduling the average overhead is described in Eq 17, where it is calculated by the sum of the cost divided by the time interval *T*.

$$C=C_{t}^{r}x_{t}^{r}+C_{t}^{o}x_{t}^{o}$$ (16)

$$C_{avg}=\frac{\sum_{i=1}^{t}C}{T}$$ (17)

#### Energy consumption

This metric might be measured by the average energy consumption, which is defined as the energy consumption ratio as listed in Eq 19, where *N* is the total number of the intervals. Additionally, the energy consumption is expressed in Eq 18.

$$Power=k\times P_{\max}+(1-k)\times P_{\max}\times u$$ (18)

Where the idle power consumption coefficient [42] *k* is equal to 0.7, and the parameter *P*_max_ represents the peak power. Additionally, *u* is based on the CPU utilization.

$$Power_{avg}=\frac{\sum_{i=1}^{n}Power}{N}$$ (19)

#### The utilization

The utilization is one of key indicators to evaluate the performance in the scheduling. The average utilization is defined as the ratio between the total CPU utilization and the total number of the intervals, as shown in Eq 20.

$$Ut_{avg}=\frac{\sum_{i=1}^{n}Ut}{N}$$ (20)

#### SLA violation

The SLA violation can be calculated by the percentage of the difference between the actual requests and allocated requests divided by the total requests, as described in Eq 21. Generally, the SLA violation might be measured by the CPU utilization [43], just in Eq 22. Then the average SLA violation is defined as the ratio between the total SLA violation and the total number of the intervals, expressed by Eq 23. In fact, the SLAV is expressed by the average SLA multiplied by the average response time, as shown in Eq 24.

SLA=Reqtotal−ReqallocateReqallocate (21)

$$sla=\frac{1}{1+e^{\left(U_{cpu}-0.8\right)}}$$ (22)

$$sla_{avg}=\frac{\sum_{i=1}^{n}sla_{i}}{N}$$ (23)

$$SLAV=sla_{avg}\times time$$ (24)

### Algorithms in comparison

To validate the ERP algorithm, we compare it with other algorithms, such as lightweight resource scaling (LS) algorithm [44], the proactive method [45], and the reactive method [46].

#### Reactive method

The traditional algorithm is scaled by the CPU utilization, obeying the simple principle by a rule-condition-action. In the experiments, the threshold is usually fixed at 0.8 or 0.2. Namely, when the utilization is higher than 0.8, the VMs would be increased. In contrast, when the utilization is lower than 0.2, the resources would be decreased.

#### Proactive method

The proactive method means that it would scale the servers up or down by the prediction technique, such as ARMA. That is, it could scale the resources up or down by the ARMA.

#### LS

The LS algorithm focuses more on the response time. When it is higher than the upper threshold the number of the VMs increases. In contrast, the number of the VMs would be scaled down. Additionally, the algorithm would shut down the spare machines by a simple predictive technique.

### Experiment results

Actually, our proposed algorithm is constructed on the performance value, which is calculated by the GRA and TOPSIS policy. In more experiments we determine that the performance threshold range is between 0.1 and 0.2. Namely, when it is greater than 0.2, we would scale up the servers, and when it is lower than 0.1 we would scale down the servers. Moreover, the performance evaluation considers multiple angles, such as maximizing the utilization, and minimizing the power consumption and the SLA violation. The results prove the effectiveness of the ERP approach.

#### The number of the servers

**Fig 5:**
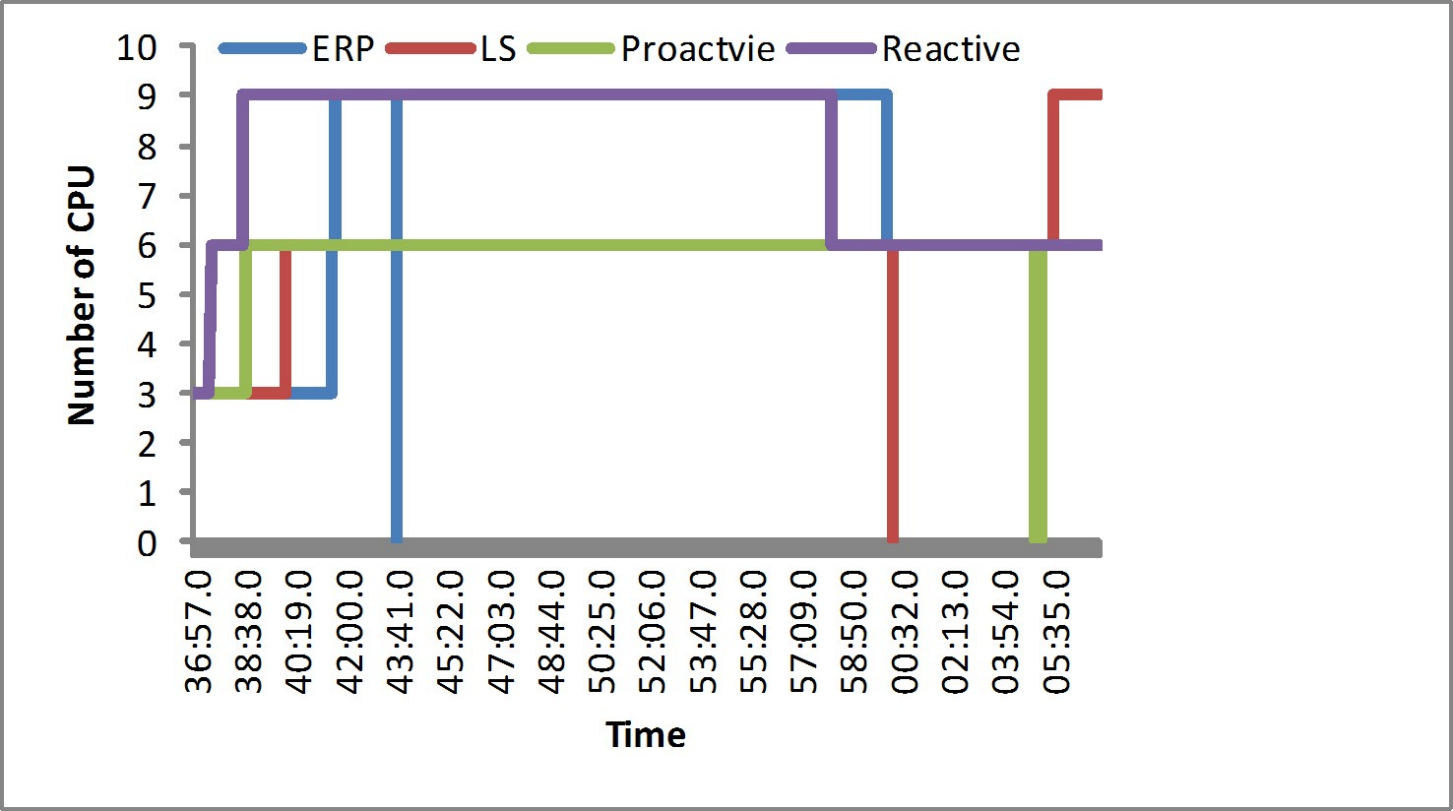
The number of CPUs in the synthetic load.

**Fig 6:**
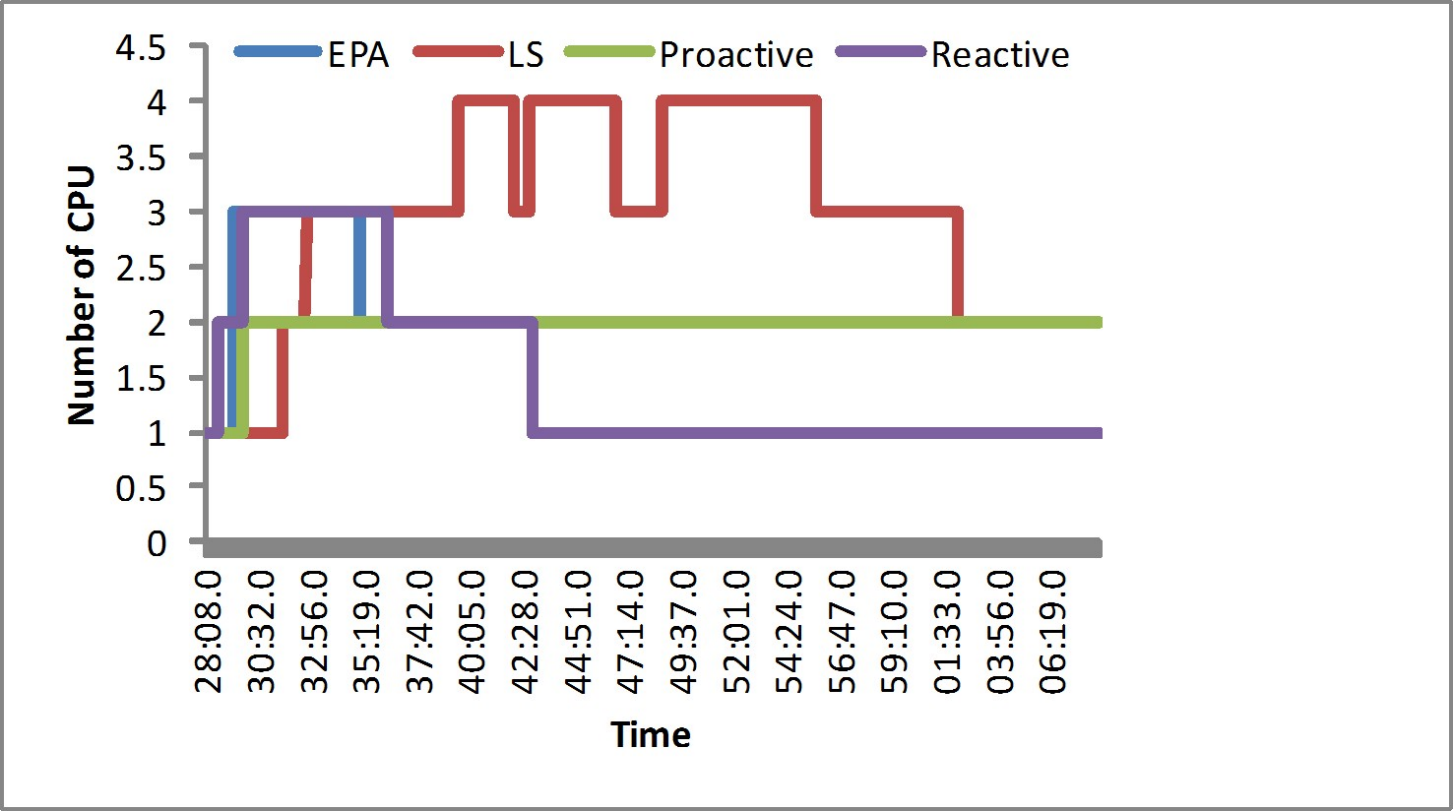
The number of CPUs in the EPA load.

**Fig 7:**
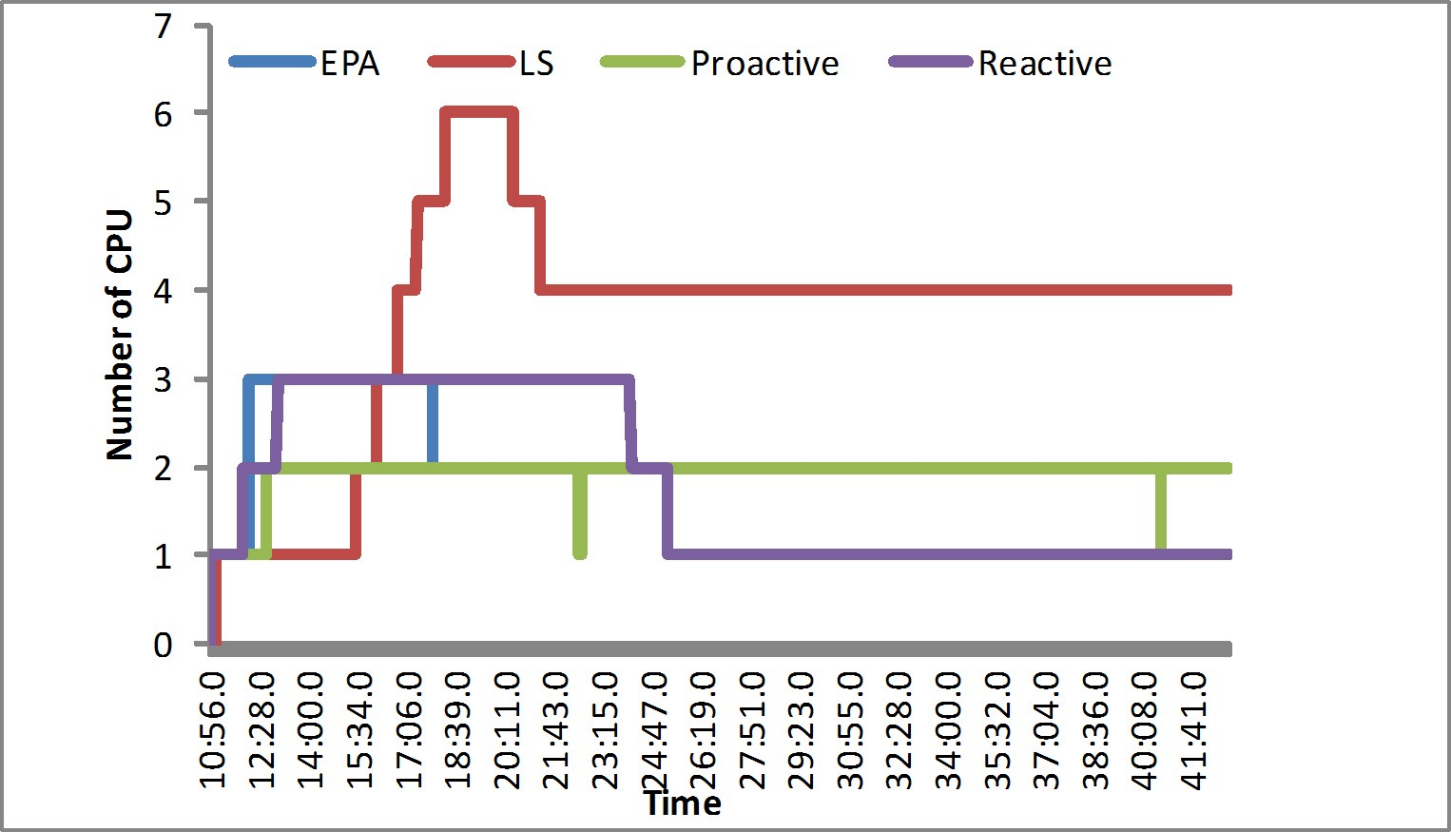
The number of CPUs in the NASA load.

In the synthetic load, the reactive algorithm puts a greater emphasis on the scalability of the servers and reacts quickly at first. The proactive algorithm would obtain the suitable number of the servers in the regular load test, and the LS algorithm spends less resources. Our proposed algorithm could occasionally occupy slightly more resources than the LS algorithm to meet the multidimension requirement in the simulated experiment at the beginning, as shown in Fig 5. In the real-world load, including EPA and NASA, our algorithm would occupy slightly more resources at first. Next it would outperform other algorithms in the normal level, as illustrated in Figs 6 and 7. We determine that the LS algorithm is unsuitable for various loads. That is, because the LS algorithm depends more on the response time. When a sudden load appears, it would increase the overhead. However, our approach has the advantage of efficiently avoiding a sudden load efficiently by reserving slightly more resources.

#### The renting cost

**Fig 8:**
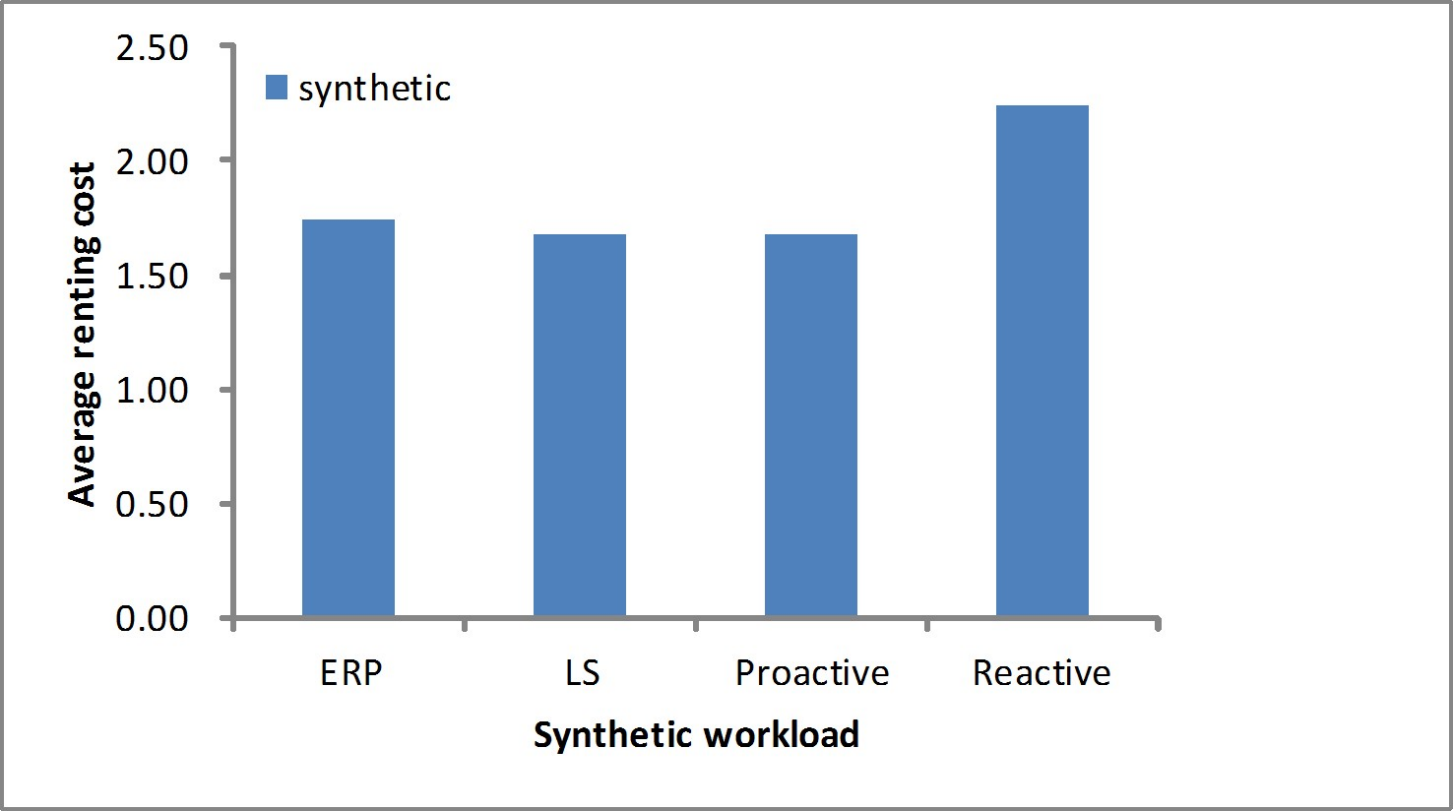
Average renting cost in the synthetic load.

**Fig 9:**
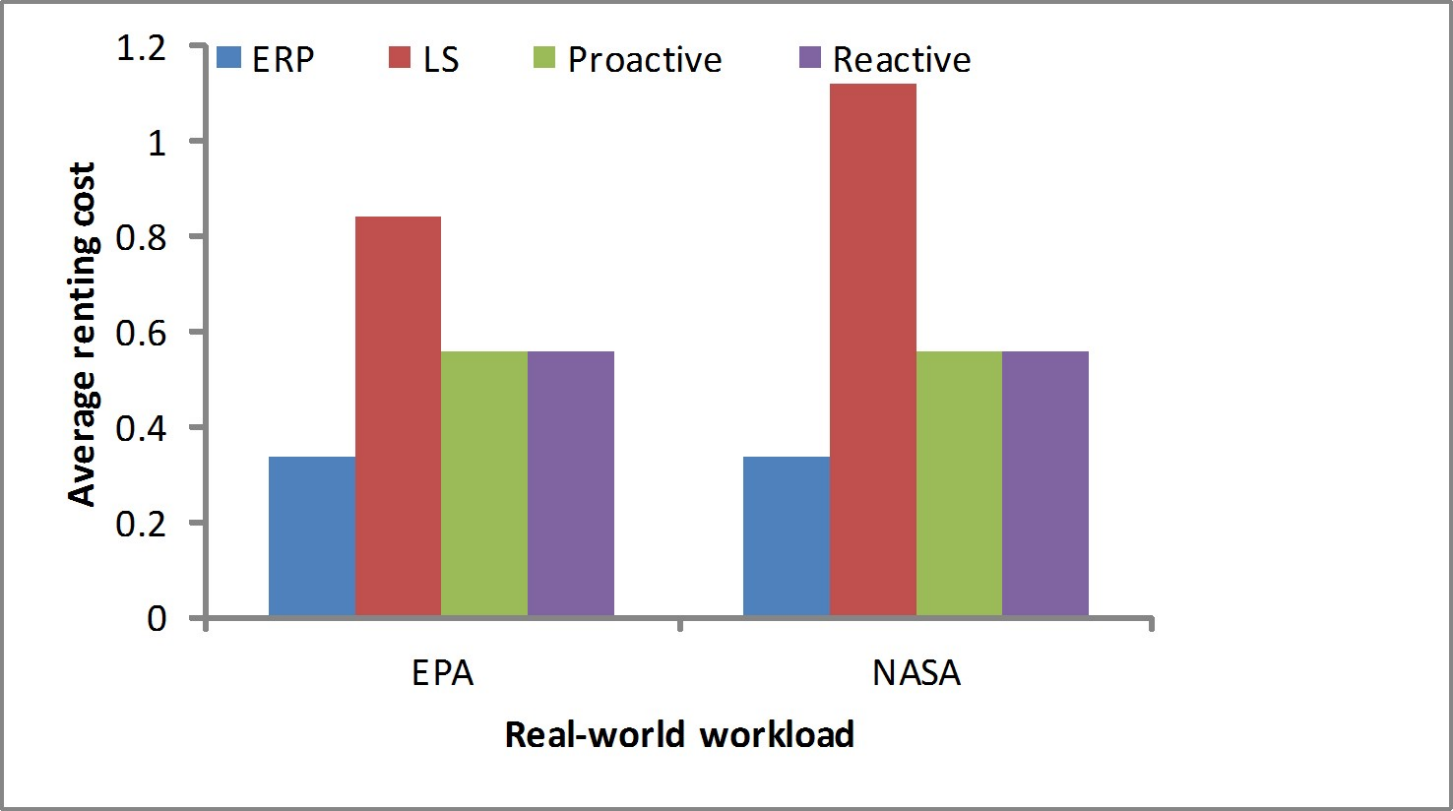
Average renting cost in the real-world workloads.

We measure the renting cost using Eq 18. As shown in Fig 8, in the synthetic load the LS algorithm puts a greater emphasis on the time to scale the resources. Namely, in the stable workload it gains the smallest average renting cost. We find that the ERP algorithm spends a slightly higher cost than the LS due to reserving few resources at first. The proactive algorithm would obtain a better result in the regular load test by the prediction. Our proposed algorithm obtains a lower cost than the reactive algorithm. As shown in Fig 9, in the real-world load we find that our proposed algorithm obtains a lower cost than the other algorithms, and the LS algorithm obtains a higher cost depending on the response time. When it appears in the sudden load, the LS algorithm would scale up the resources more quickly, which makes the occupied resources greater than in other algorithms.

#### Resource utilization

**Fig 10:**
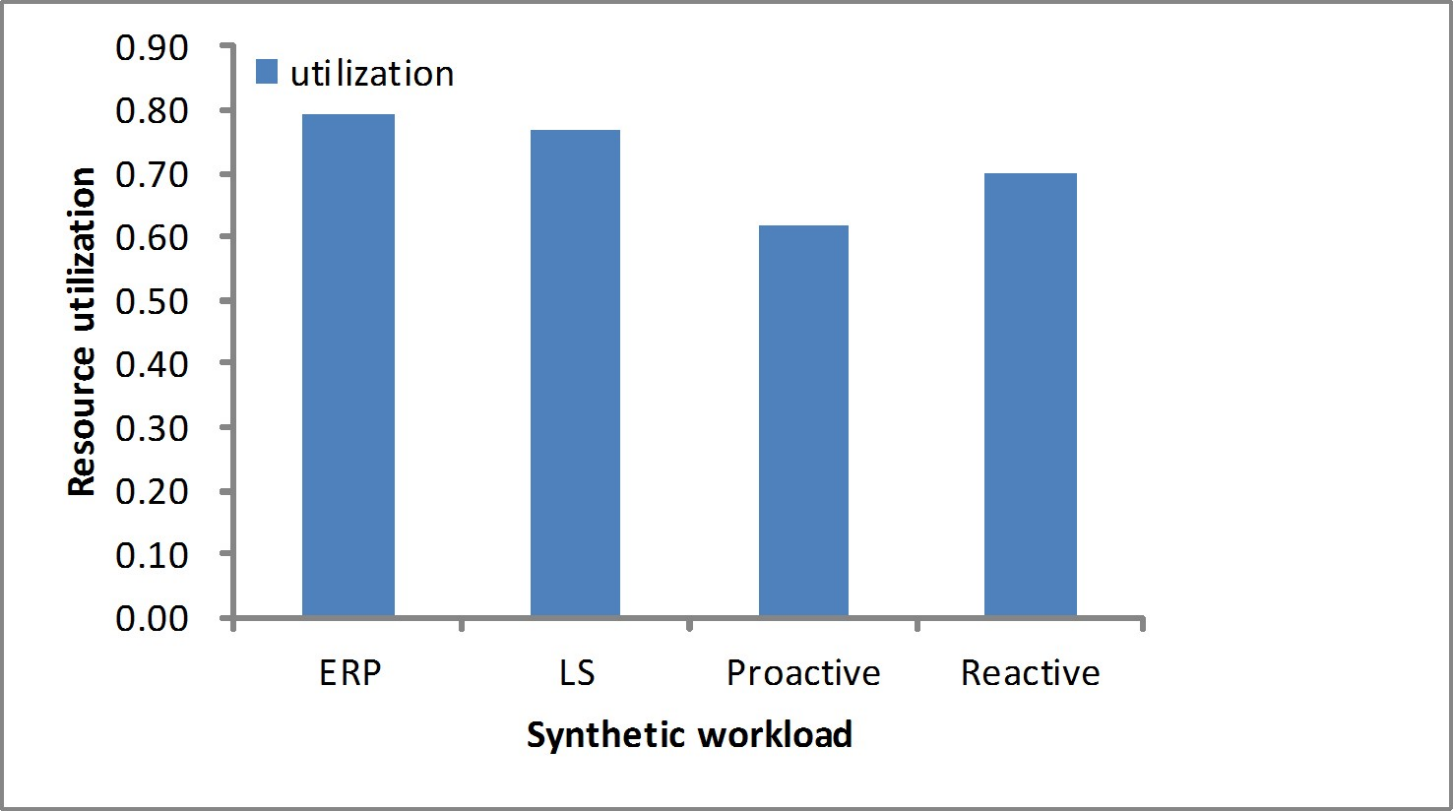
Average resource utilization in the synthetic workload.

**Fig 11:**
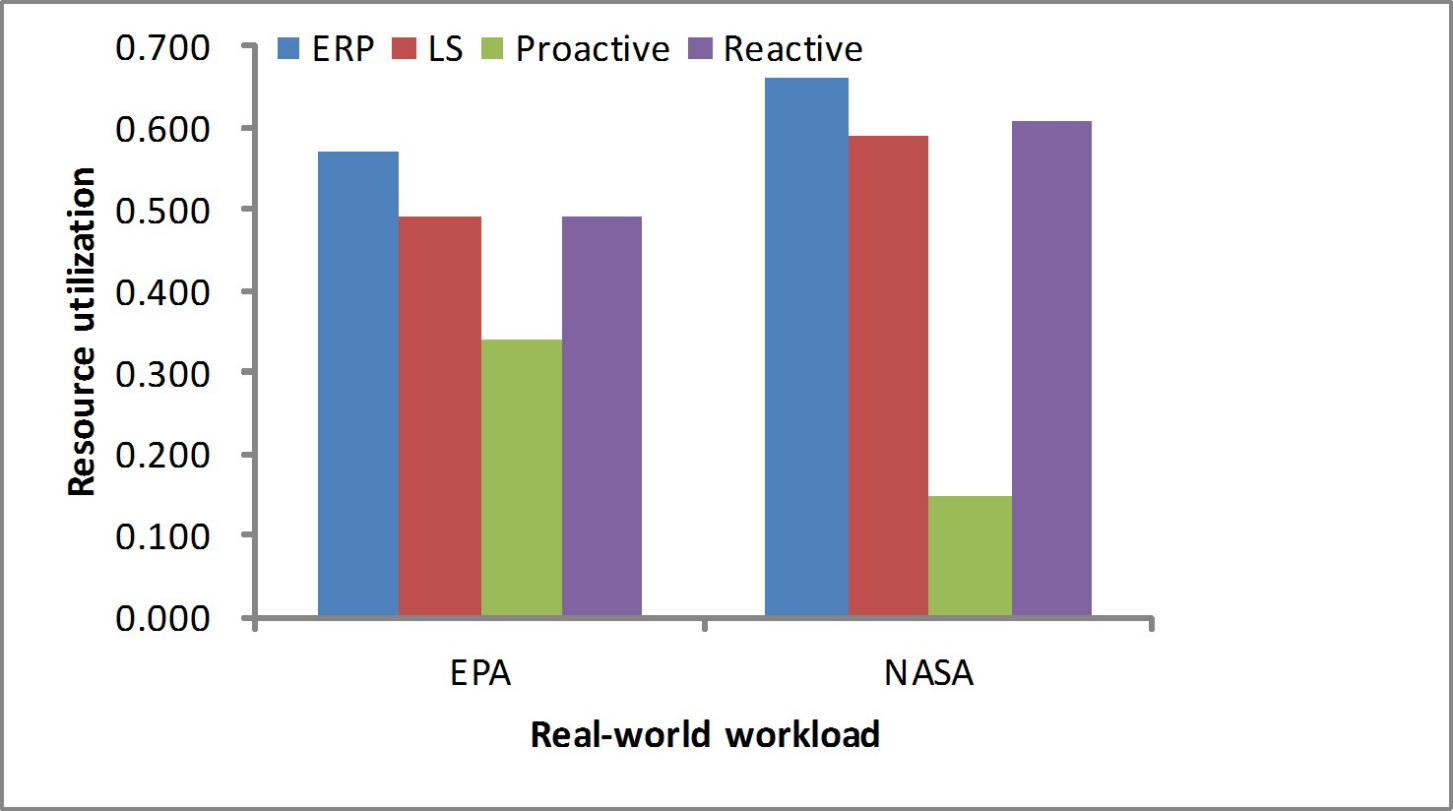
Average resource utilization in the real-world workloads.

We measure the average resource utilization based on Eq 20. Figs 10 and 11 show the CPU utilization during the experiments under different workloads, including the synthetic load and real-world loads. In these experiments, we determine that the proposed approach utilizes the resources more fully, which is depicted in Figs 10 and 11. In the experiments the ERP method consumes slightly more resources at first and simultaneously guarantees a lower SLA violation rate. Additionally, it releases the servers by the WMA prediction by guaranteeing the performance in the varying workloads. We see that no resource utilization is higher than 100%, which proves that our approach efficiently reduces the underprovisioning state.

#### Response time

**Fig 12:**
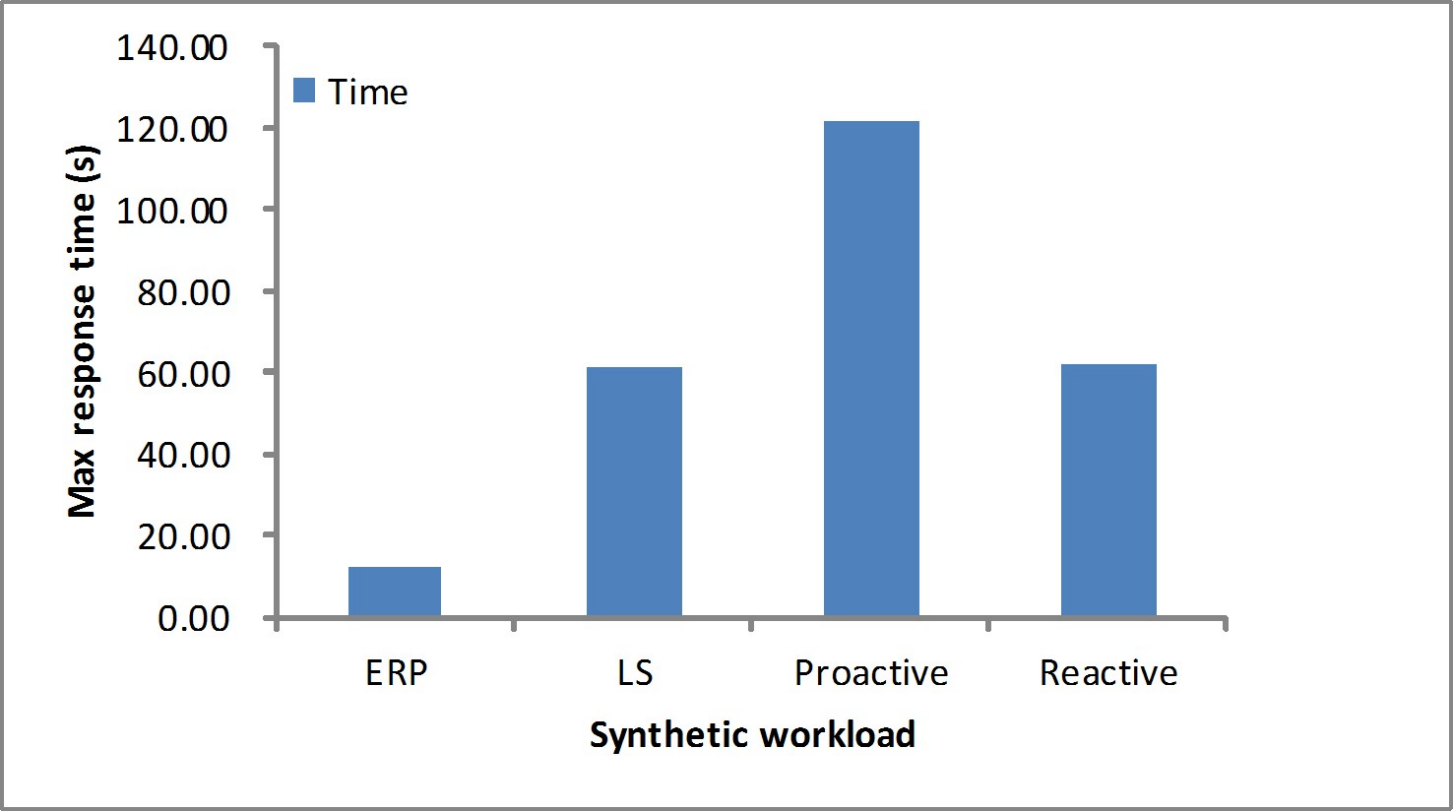
Max response time in the synthetic workload.

**Fig 13:**
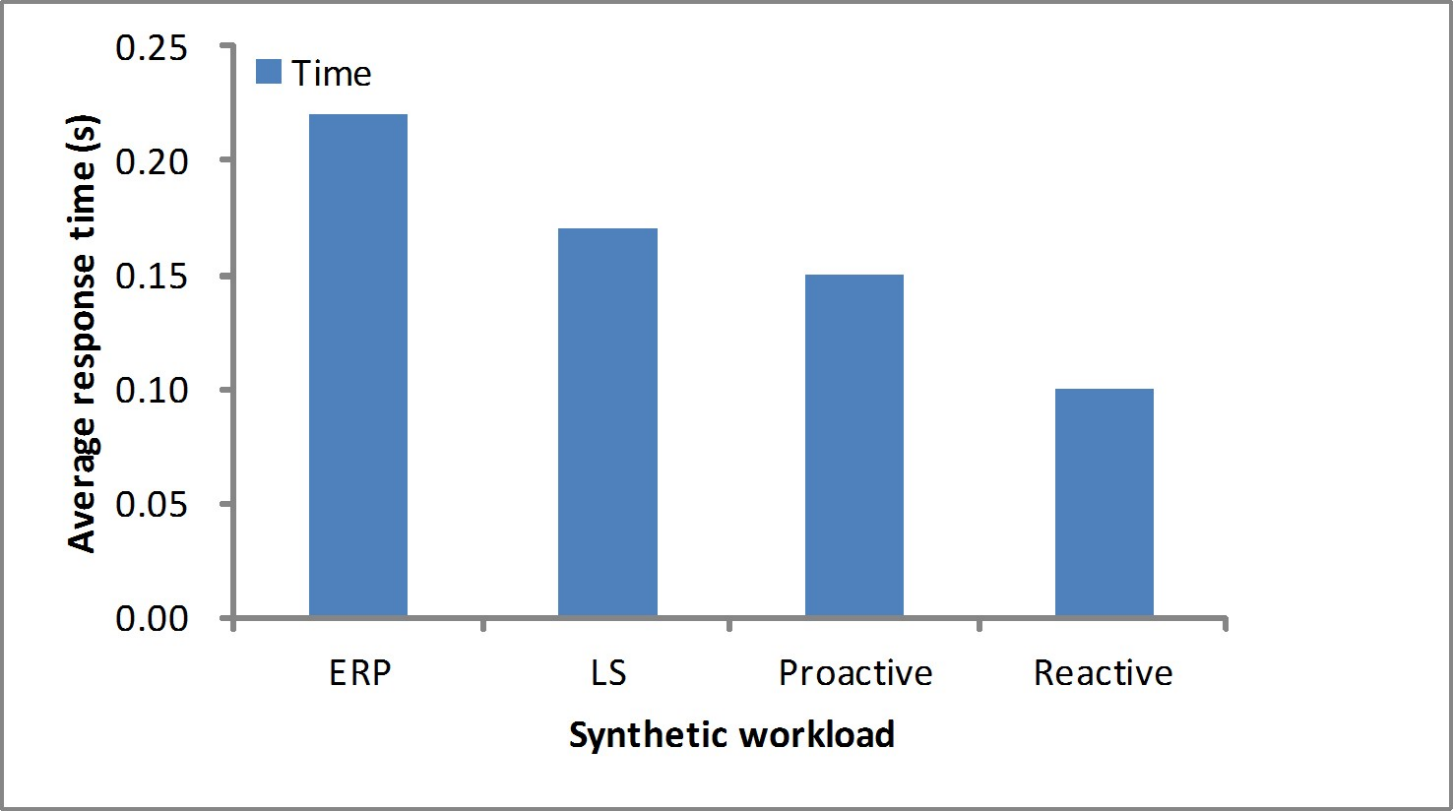
Average response time in the synthetic workload.

**Fig 14:**
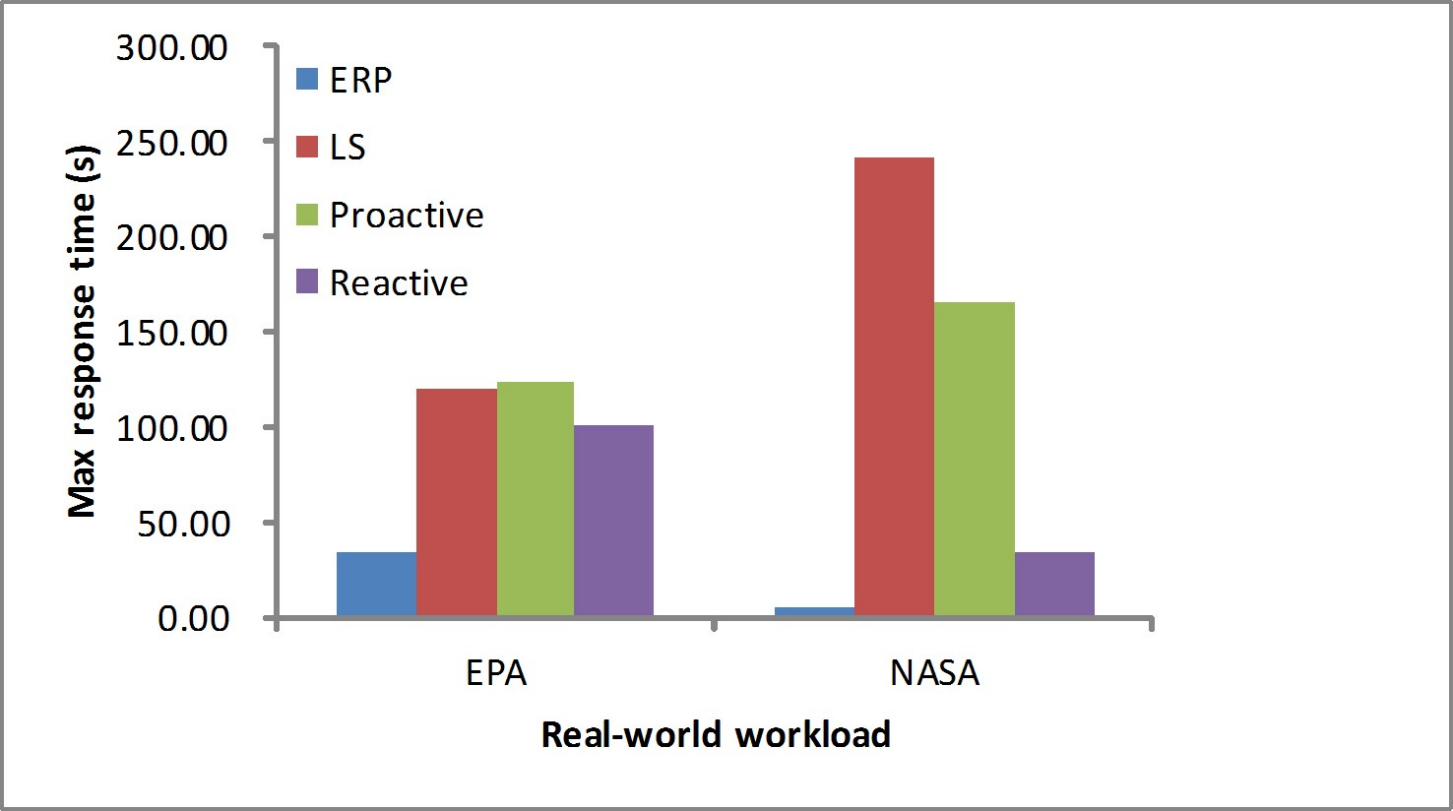
Max response time in the real-world workloads.

**Fig 15:**
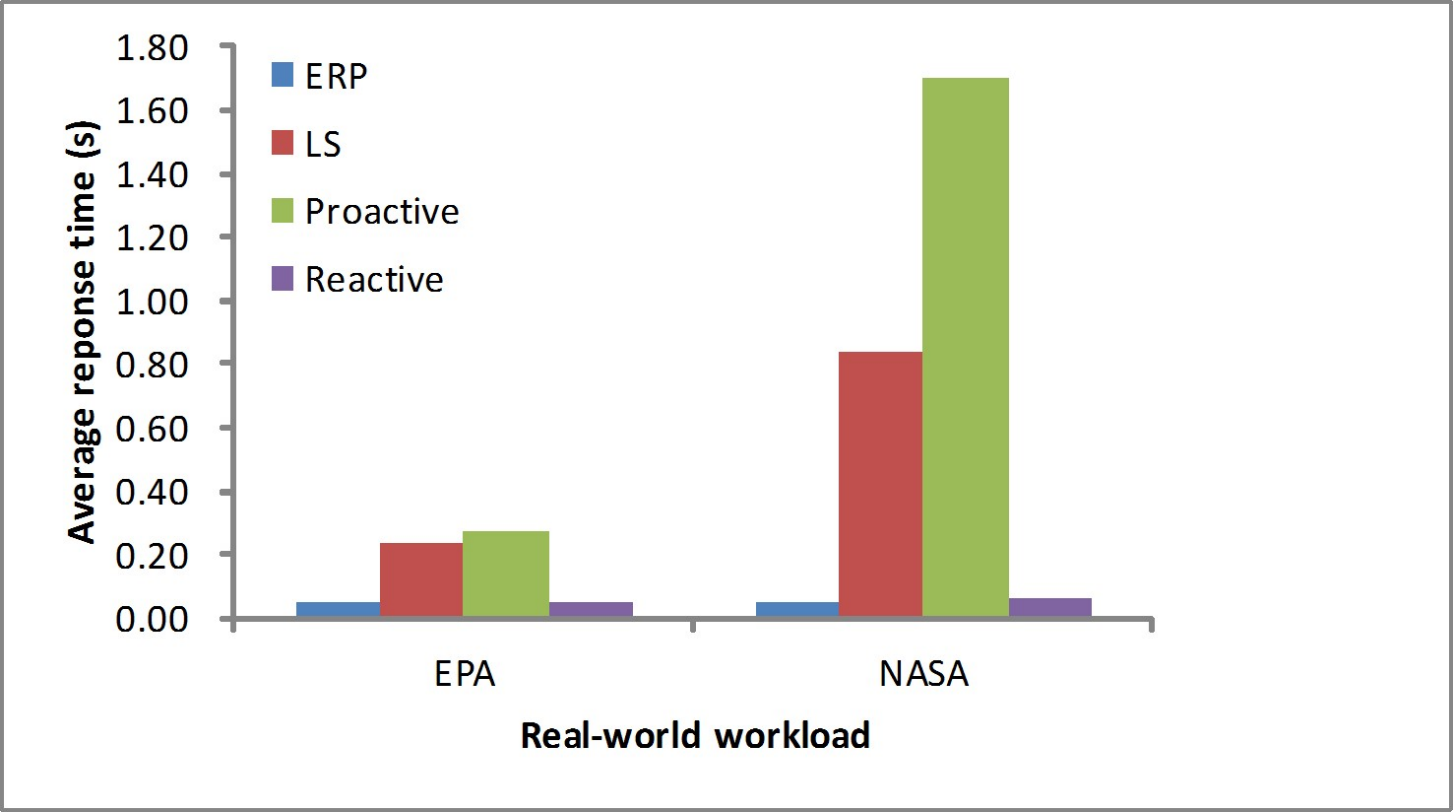
Average response time in the real-world workloads.

The response time is another performance metric that needs to be studied. As depicted in Figs 12 and 13, in the synthetic workload, when considering the maximum response time, we determine that our proposed algorithm obtains a quicker response than the other algorithms by reserving few resources at first. For the average response time, these algorithms are in the acceptable level at the stable workload. As depicted in Figs 14 and 15, in the real-world loads we find that our algorithm presents a lower maximum or average response time than others by reserving slightly more resources at first, while the LS algorithm obtains slightly higher time due to a longer monitoring time. Additionally, it is unfit for the sudden load. In the NASA load the variable workload leads to inaccurate prediction values, so the proactive algorithm obtains a longer average response time.

#### SLA violation

**Fig 16:**
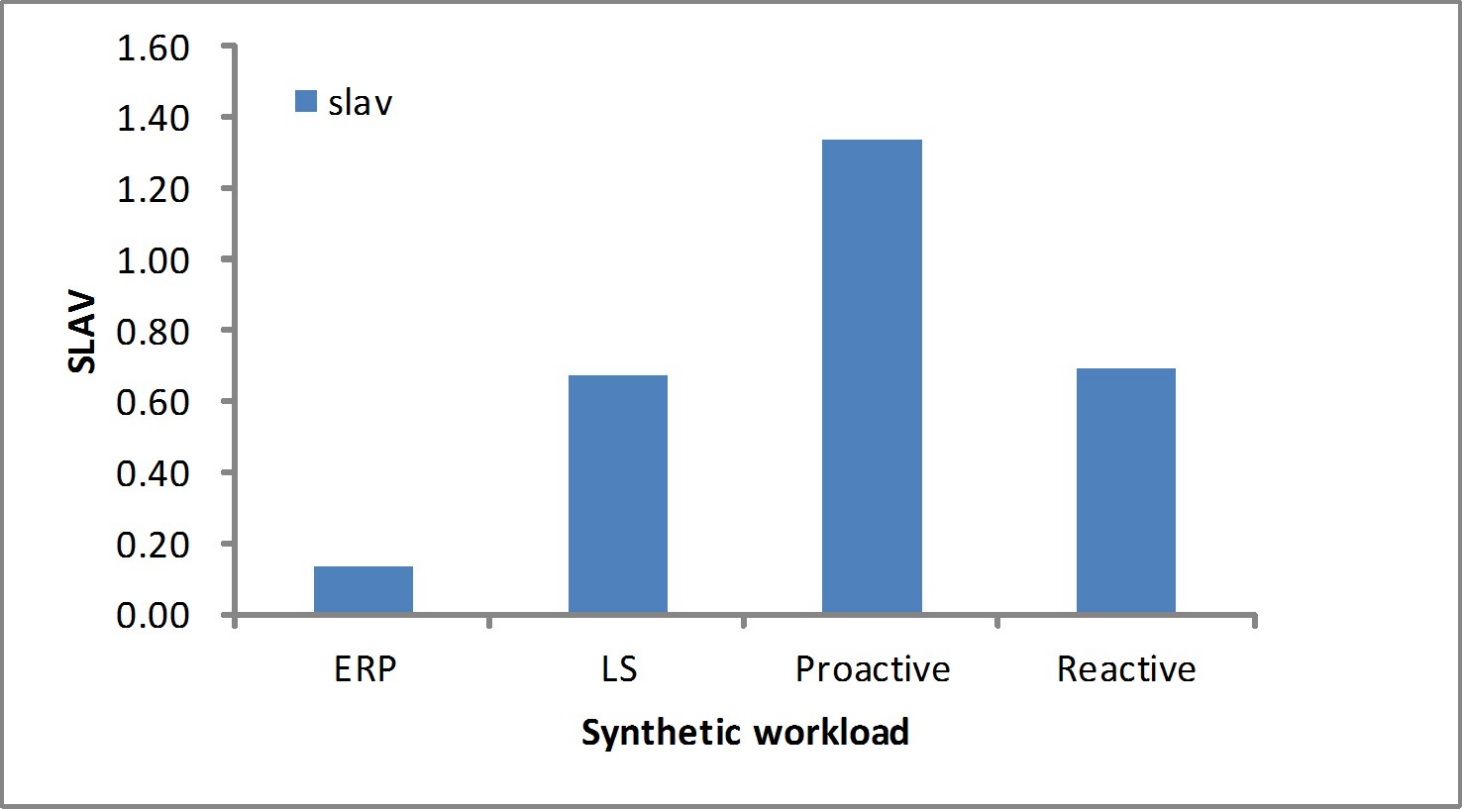
SLAV in the synthetic workload.

**Fig 17:**
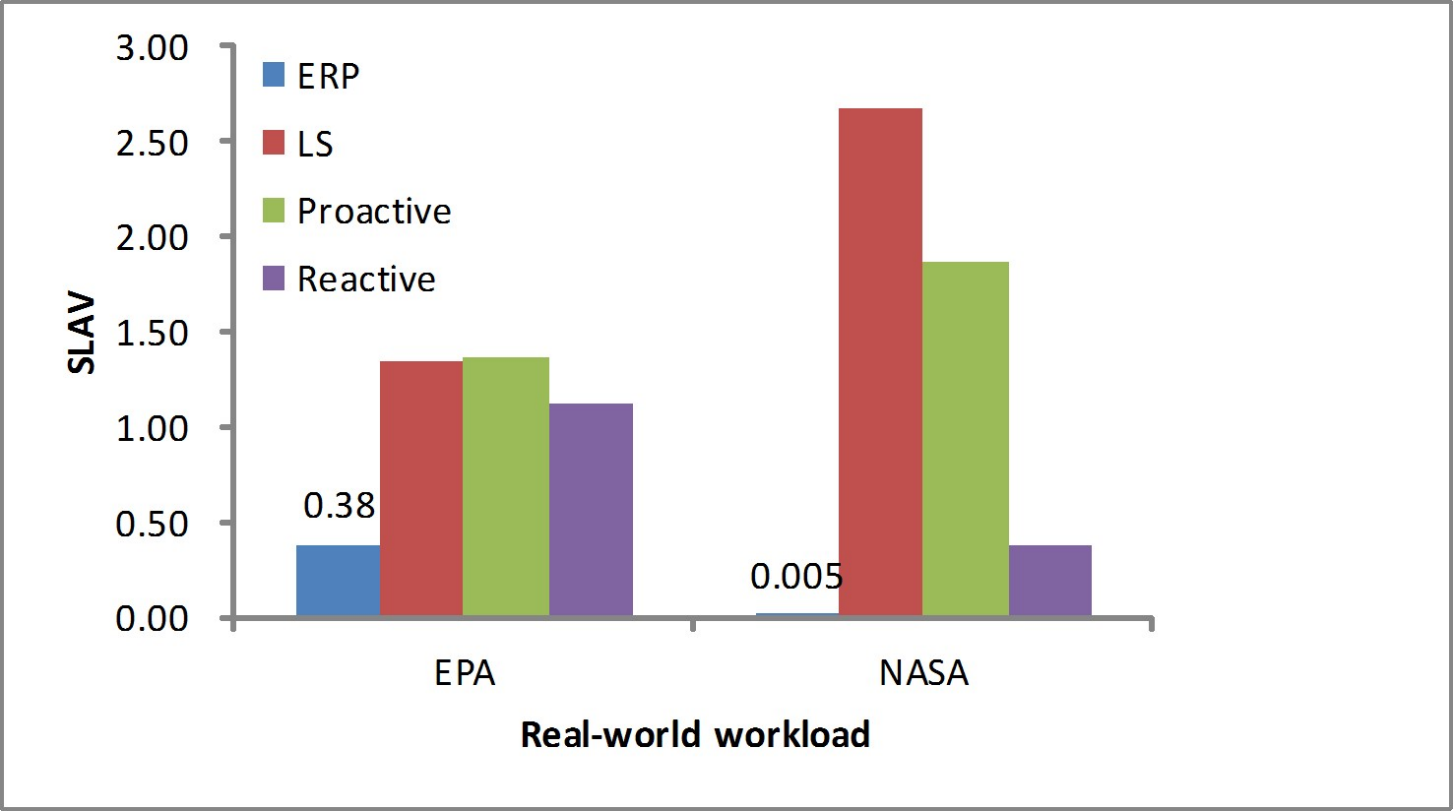
SLAV in the real-world workloads.

We measured the SLA violation based on Eq 24. As shown in Figs 16 and 17, in the workloads our algorithm presents a lower SLA violation ratio than the other algorithms. In addition, the error rate is another metric that evaluates the performance. As listed in Table 5, we see that our algorithm produces a slightly lower error ratio and efficiently avoids the sudden load.

**Table 5:** The error analysis in the experiments.

Type	ERP (error%)	LS (error%)	Proactive (error%)	Reactive (error%)
Synthetic	0	0	0	0
EPA	0	0.01%	0.02%	0
NASA	0	1.22%	22.50%	0

#### Average energy consumption

**Fig 18:**
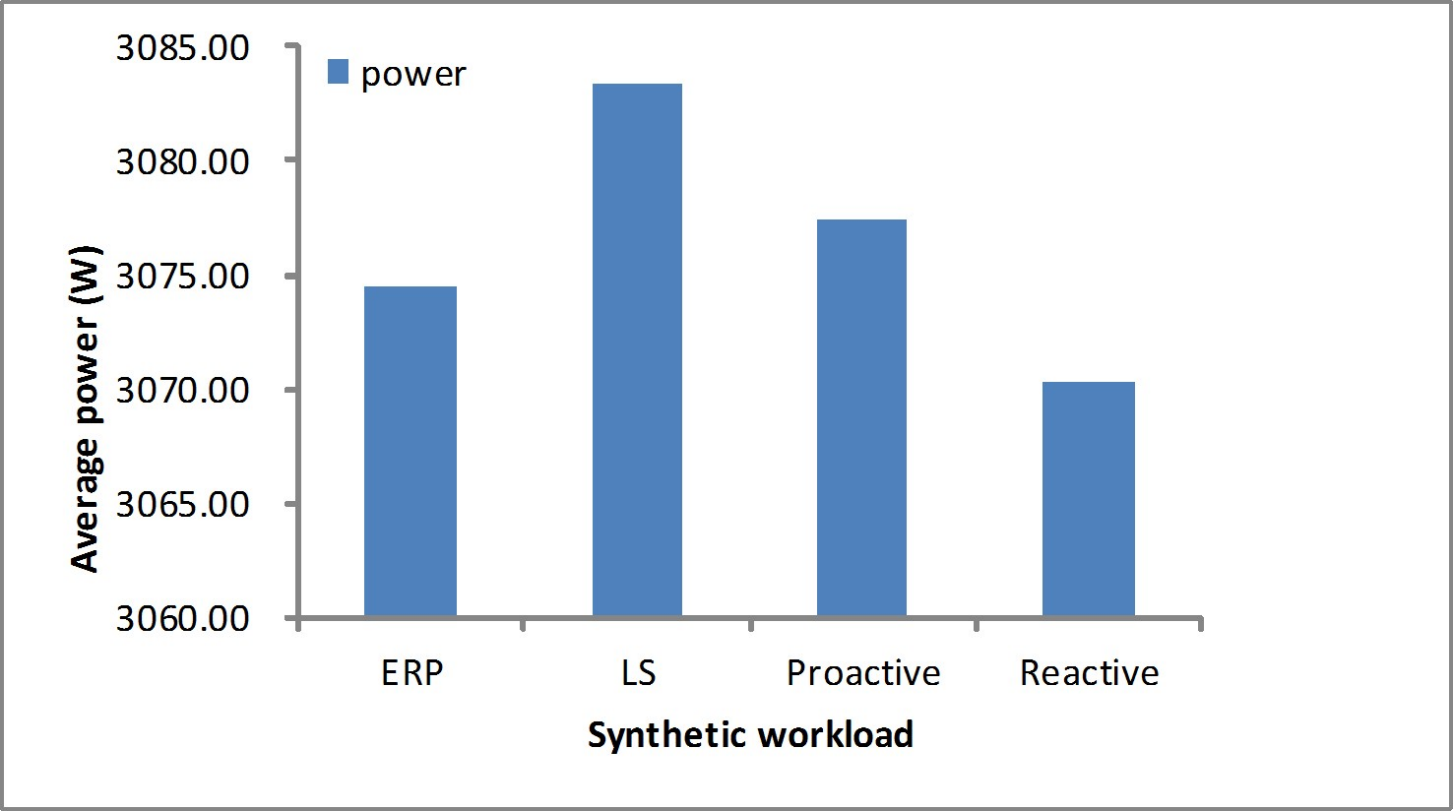
Energy consumption in the synthetic workload.

**Fig 19:**
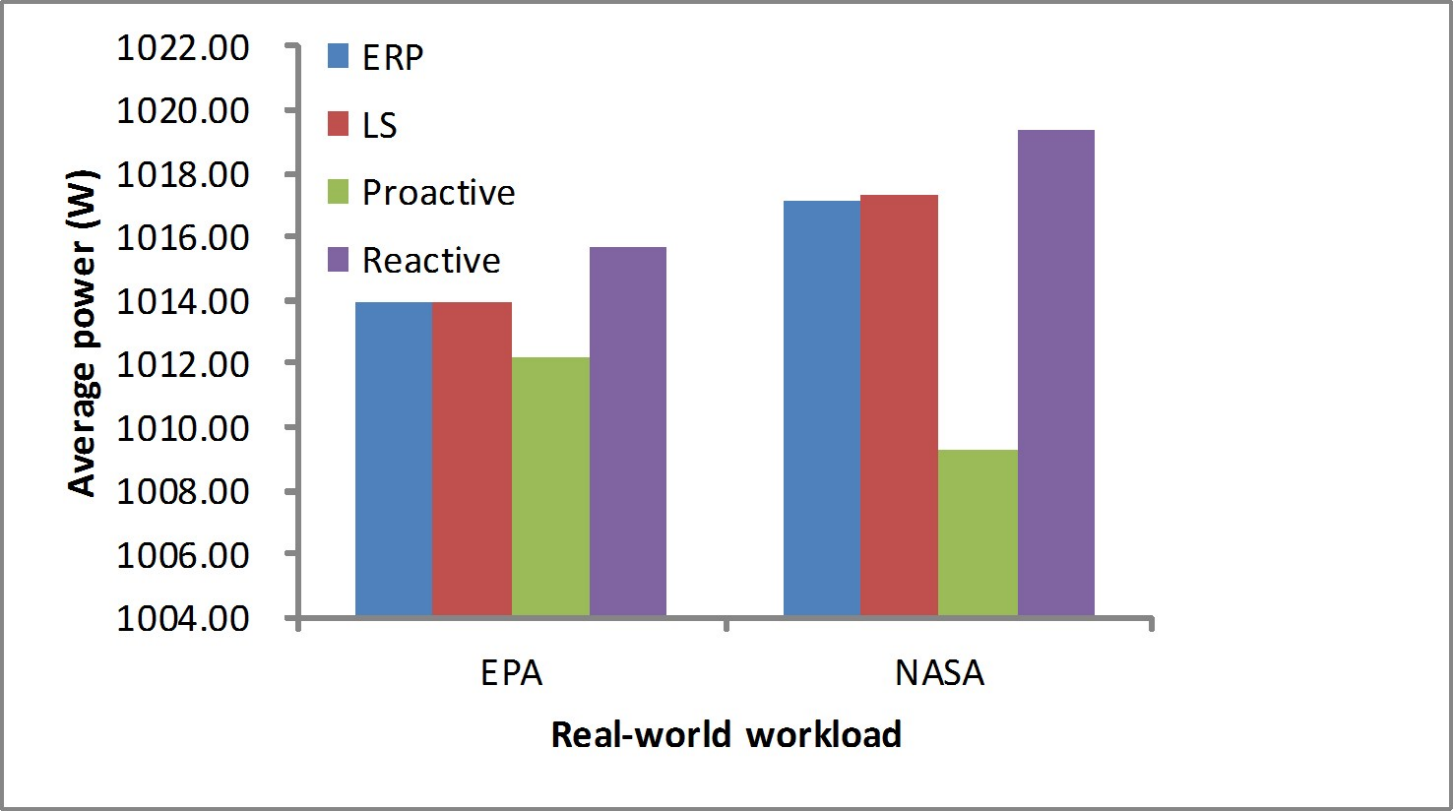
Energy consumption in the real-world workloads.

We measure the average energy consumption based on Eq 19. As shown in Fig 18, in the synthetic load our algorithm achieves a lower power than the LS and proactive algorithms. Since it is in the stable workload, the reactive algorithm obtains a better result than the other algorithms only by the utilization. As shown in Fig 19, in real-world loads the proposed algorithm presents a lower power than the LS and reactive algorithm. The proactive method consumes less energy consumption than others, but it cannot meet the demand due to the inaccurate prediction. This is because that it achieves a higher error rate in Table 5.

## Conclusion

Traditional elasticity is often used as a reactive method, which is implemented by the rule-condition-action. However, it would be a better strategy to combine this with the prediction. In this paper, we present an elastic strategy that increases or decreases the resources by the performance threshold in a flexible manner. To further elaborate, the ERP approach makes the following contributions. First, we present the performance threshold depending on the CPU and the memory. By this, we could flexibly scale the resources up or down. This solves the issue of deciding a suitable threshold on multiple elements. Second, we propose an SUS algorithm that implements the fine-grained scaling in the PM-Level or VM-Level to increase the resources flexibly. This solves the issue of an elastic scaling strategy from different granularities to reduce the SLA violation and response time. Third, combining this with the WMA prediction we propose the SDS algorithm to scale down the servers. Then we would shut down the spare machines to save energy consumption. This solves the issue of effectively saving the overheads. Finally, we evaluate the proposed ERP approach in the simulated and real-world workloads. The results show that the ERP method improves the utilization, minimizes the renting cost, saves the energy consumption and gives a quicker response time.

In fact, we implement the scaling approach on the premise of regarding the servers as the available resources. However, no cloud provider offers unlimited resources, except for Google and Amazon. Thus, a further study should be made on some aspects. First, it is necessary to find an effective way to minimize the renting cost by reserving some available resources in advance. However, more servers would be wasted by reserving too many resources. Therefore, it is necessary to balance the reserved plan and the on-demand plan. Second, from the perspective of minimizing the energy consumption, a reasonable dynamical provisioning approach might efficiently consolidate the available resources by the migration technique. Then in the future it will be necessary to explore the dynamical provisioning approach in the complex workloads. Perhaps some typical types of the workflow would be an interesting extension in the future.

## Supporting information

S1 Table Synthetic load or real-world workloads by Jmeter. The synthetic workload is generated by Jmeter, as is the simulated real workload, such as the EPA and the NASA.
(XLSX)

S2 Table The number of CPUs in the synthetic load. The number of CPU in the synthetic load and real-world load is monitored by Jmeter plugin.
(XLSX)

S3 Table The logs in the synthetic load. The logs record the power, the SLA and the utilization.
(XLSX)

S4 Table The logs in the EPA load. The logs record the power, the SLA and the utilization.
(XLSX)

S5 Table The logs in the NASA load. The logs record the power and the SLA. Additionally, the utilization is evaluated in the workloads.
(XLSX)

S1 Performance Evaluation The SLA violation and energy consumption are evaluated in the synthetic load and real-world workloads. (XLSX)
